# A gossypol derivative effectively protects against Zika and dengue virus infection without toxicity

**DOI:** 10.1186/s12915-022-01344-w

**Published:** 2022-06-15

**Authors:** Yaning Gao, Wanbo Tai, Xinyi Wang, Shibo Jiang, Asim K. Debnath, Lanying Du, Shizhong Chen

**Affiliations:** 1 Department of Natural Medicines, School of Pharmaceutical Sciences, Peking University, Beijing, 100191 China; 2 Lindsley F. Kimball Research Institute, New York Blood Center, New York, NY 10065 USA; 3 Key Laboratory of Medical Molecular Virology (MOE/NHC/CAMS), School of Basic Medical Sciences, Fudan University, Shanghai, 200032 China

**Keywords:** Flavivirus, Zika virus, Dengue virus, Antiviral agent, In vitro inhibition, In vivo protection, Toxicity

## Abstract

**Background:**

Zika virus (ZIKV) and dengue virus (DENV) cause microcephaly and dengue hemorrhagic fever, respectively, leading to severe problems. No effective antiviral agents are approved against infections of these flaviviruses, calling for the need to develop potent therapeutics. We previously identified gossypol as an effective inhibitor against ZIKV and DENV infections, but this compound is toxic and not suitable for in vivo treatment.

**Results:**

In this study, we showed that gossypol derivative ST087010 exhibited potent and broad-spectrum in vitro inhibitory activity against infections of at least ten ZIKV strains isolated from different hosts, time periods, and countries, as well as DENV-1-4 serotypes, and significantly reduced cytotoxicity compared to gossypol. It presented broad-spectrum in vivo protective efficacy, protecting ZIKV-infected *Ifnar1*^*−/−*^ mice from lethal challenge, with increased survival and reduced weight loss. *Ifnar1*^*−/−*^ mice treated with this gossypol derivative decreased viral titers in various tissues, including the brain and testis, after infection with ZIKV at different human isolates. Moreover, ST087010 potently blocked ZIKV vertical transmission in pregnant *Ifnar1*^*−/−*^ mice, preventing ZIKV-caused fetal death, and it was safe for pregnant mice and their pups. It also protected DENV-2-challenged *Ifnar1*^*−/−*^ mice against viral replication by reducing the viral titers in the brain, kidney, heart, and sera.

**Conclusions:**

Overall, our data indicate the potential for further development of this gossypol derivative as an effective and safe broad-spectrum therapeutic agent to treat ZIKV and DENV diseases.

**Supplementary Information:**

The online version contains supplementary material available at 10.1186/s12915-022-01344-w.

## Background

Zika virus (ZIKV), a mosquito-borne flavivirus [1], has acquired the ability to infect humans through other routes, such as vertical transmission [2–5], leading to microcephaly [6–8], fetal damage, and fetal or pup death [9–11]. ZIKV infection may also cause damage to the testis and uteri [12–15]. ZIKV cases increased rapidly during 2015 and 2016. According to the statistics from the Pan American Health Organization (PAHO), there were at least 515,000 suspected cases of ZIKV local infection in the Americas from 2015 to 2016 and 168,000 confirmed cases as of November 2, 2016 [16]. There were less ZIKV cases since then. The symptoms of ZIKV infection mainly include maculopapular rash, fever, joint pain or arthritis, non-suppurative conjunctivitis, myalgia, headache, eye socket pain, edema, and vomiting [17].

ZIKV is a single-stranded RNA virus [18]. The ZIKV genome encodes three structural proteins, including capsid (C), pre-membrane/membrane (prM/M), and envelope (E), in addition to seven nonstructural (NS) proteins, such as NS1, NS2A, NS2B, NS3, NS4A, NS4B, and NS5 [19, 20]. The E protein, which consists of domain I (EDI), EDII, and EDIII, a fusion loop, and a stalk region, plays a key role in viral entry and membrane fusion [21, 22]. The NS2B-NS3 protease complex is composed of NS2B cofactor and NS3 protease domain, whereas the NS2B cofactor is essential for recognizing host cell substrates [23–25]. The NS2B-NS3 protease complex is responsible for ZIKV protein maturation via cleaving the polyprotein precursor to generate fully functional proteins, determining its critical role as an important antiviral drug target [23, 24, 26, 27].

The life cycle of ZIKV can be briefly summarized as follows [28]. Host cell membrane receptors bind the E protein of the mature ZIKV virion, triggering endocytosis. The acidic environment of the endosome induces fusion of the host endosome membrane with the viral envelope, and the release of the RNA genome. The RNA is translated into a polyprotein complex, which is cleaved by the host and viral proteases (including NS2B-NS3) in the endoplasmic reticulum (ER) lumen and cytoplasm, respectively. Following translation, a replication complex is assembled and associated with virus-induced membranes where viral replication takes place. The methylated (+) ssRNA, C, E, and prM proteins are assembled to form immature virions in the ER. The immature virions bud out of the ER into the Golgi apparatus, and they then mature in the trans-Golgi network that are released by exocytosis.

ZIKV is closely related to the dengue virus (DENV), another member in the family *Flaviviridae* and genus *Flavivirus* [29]. DENV has four distinct serotypes (DENV-1-4) whose genomes encode structural and NS proteins similar to those of ZIKV [30]. Different from ZIKV, however, DENV infection may cause dengue fever (DF), dengue hemorrhagic fever (DHF), and even dengue shock syndrome (DSS), leading to an increase in dengue cases and severe problems [31, 32]. Primary DNEV infection from the same serotype generally causes mild symptoms; however, subsequent infections from different DENV serotypes may result in antibody-dependent enhancement with the potential to develop DF, DHF, or DSS. DENV is a global public health threat, and about two-thirds of the world population are at risk of DENV infection, causing around 390 million infections annually [33, 34].

Although many vaccine candidates have been developed against ZIKV or DENV infection, most of them are still in the preclinical development [35, 36]. A live-attenuated chimeric yellow fever 17D-tetravalent dengue vaccine (CYD-TDV, Dengvaxia) has been licensed for clinical use against DENV infection in individuals over 9 years old in several dengue-endemic countries [37]; however, no vaccines are approved for use in humans against ZIKV infection [36].

As two important members of the *Flaviviridae* family, ZIKV and DENV are transmitted through arthropods and share the same transmission vector [38], but they cause different diseases [39, 40]. Currently, no antiviral agents are approved against ZIKV and DENV infections; therefore, safe, effective, and broad-spectrum therapeutics are continuously needed to treat diseases caused by these flaviviruses. We previously identified a compound, gossypol, as an anti-ZIKV and anti-DENV therapeutic agent [41]. Gossypol is a polyphenolic aldehyde extracted from cottonseed that has been used as a male contraception [42]. However, gossypol is generally toxic, which is potentially associated with the aldehyde groups [43–45]. Accordingly, efforts have been made to explore the possibility of using derivatives, or analogs, of gossypol as alternative antiviral agents, in which the aldehyde groups are altered [46]. As a result, a number of gossypol derivatives, e.g., Schiff’ bases, esters, and ethers, have been characterized, many of which were reviewed previously [47], showing antimalarial, antiparasitic, anticancer, and antiviral activities. This calls for the identification of gossypol derivatives with potent antiviral efficacy against ZIKV and DENV infections, but without the toxic consequences.

Therefore, in this study, we screened a series of gossypol derivatives for their anti-ZIKV and anti-DENV activity and potential cytotoxicity. We identified five “hit” compounds with inhibitory activity, but reduced toxicity. Among these derivatives, ST087010 demonstrated strong potency against divergent ZIKV and DENV infections in vitro, but low toxicity both in vitro and in vivo. We also evaluated its broad-spectrum in vivo protection against challenge of different ZIKV strains and DENV-2 in susceptible interferon-α/β receptor (IFNAR)-deficient (*Ifnar1*^***−****/****−***^) mice.

## Results

### Identification of gossypol derivatives with potent inhibitory activity against ZIKV infection, but low cytotoxicity in vitro

**Fig. 1:**
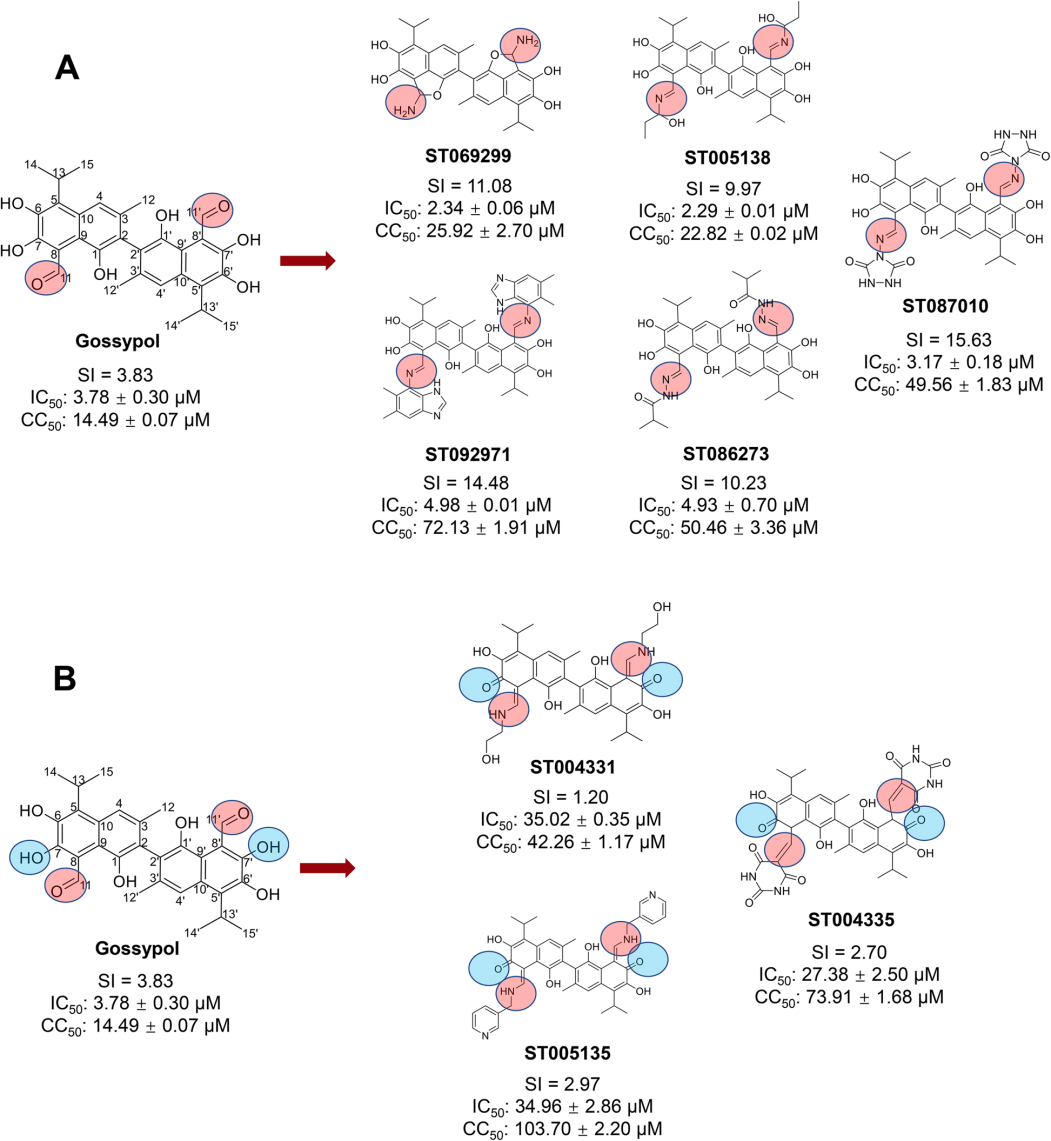
Structure, anti-Zika virus (ZIKV) activity, and cytotoxicity of gossypol derivatives. The experiments were performed on Vero E6 cells, and the cytotoxicity of gossypol derivatives in this cell line is expressed as 50% cytotoxic concentration (CC_50_). The inhibitory activity of gossypol derivatives against infection of ZIKV human strain PAN2016 (2016/Panama) is expressed as 50% inhibitory concentration (IC_50_). IC_50_, CC_50_, and selectivity index (SI) values of gossypol and each of its derivatives are shown in the figure. The red shading (**A**) indicates that the aldehyde groups on the C8 and C8’ positions of gossypol are replaced by other groups, whereas the blue shading (**B**) shows that the free hydroxyl groups on the C7 and C7’ positions of gossypol core are modified. The data are presented as the mean ± standard error of the mean (s.e.m.) of duplicate wells. The experiments were repeated twice with similar results

Sixteen gossypol derivatives covalently coupled with different chemical groups were assessed for their inhibitory activities against ZIKV infection using a plaque assay (Additional file 1: Table S1). Five derivatives, ST069299, ST005138, ST087010, ST092971, and ST086273, which showed stronger inhibitory activity than other derivatives, were identified as “hit” gossypol derivatives (Fig. 1A, Additional file 1: Table S1). Inhibitory activity of “hit” gossypol derivatives against ZIKV infection is expressed as 50% inhibitory concentration (IC_50_). All of the “hit” derivatives were able to effectively inhibit infection of ZIKV (human strain PAN2016) with the IC_50_ values ranging from 2.29 to 4.98 μM. The cytotoxicity of these derivatives was investigated by a cell-based assay in Vero E6 cells, and their 50% cytotoxic concentration (CC_50_) values ranged from 22.82 to 72.13 μM (Fig. 1A). Apparently, the cytotoxicity of all five derivatives was less than that of gossypol, among which ST069299, ST005138, and ST087010 had increased inhibitory activity against ZIKV infection, as compared to gossypol. Structural analysis indicated that all five compounds are derivatives of gossypol with the substitution of C8 and C8’ aldehyde groups (Fig. 1A), suggesting that the cytotoxicity of gossypol may be related to the C8 and C8’ aldehyde groups and that replacement of these aldehyde groups in gossypol with other groups resulted in reduced cytotoxicity.

To further elucidate the relationship between the structure of gossypol and its inhibitory activity, we analyzed the structure of three gossypol derivatives with significantly reduced inhibitory activity. The results showed that these three compounds are derivatives in which the C7 and C7’ hydroxyl groups on the gossypol core were substituted and that their cytotoxicity was also reduced (Fig. 1B). These data suggest that the free hydroxyl groups at the C7 and C7’ positions on the gossypol core are necessary for gossypol to exert its antiviral activity. Thus, modification of these hydroxyl groups resulted in reduced anti-ZIKV gossypol activity, as well as decreased cytotoxicity. Structural analysis of these compounds revealed that the aldehyde groups at the C8 and C8’ positions of these three gossypol derivatives were also replaced by other groups, suggesting that the cytotoxicity of gossypol is related to the aldehyde groups at the C8 and C8’ positions.

**Fig. 2:**
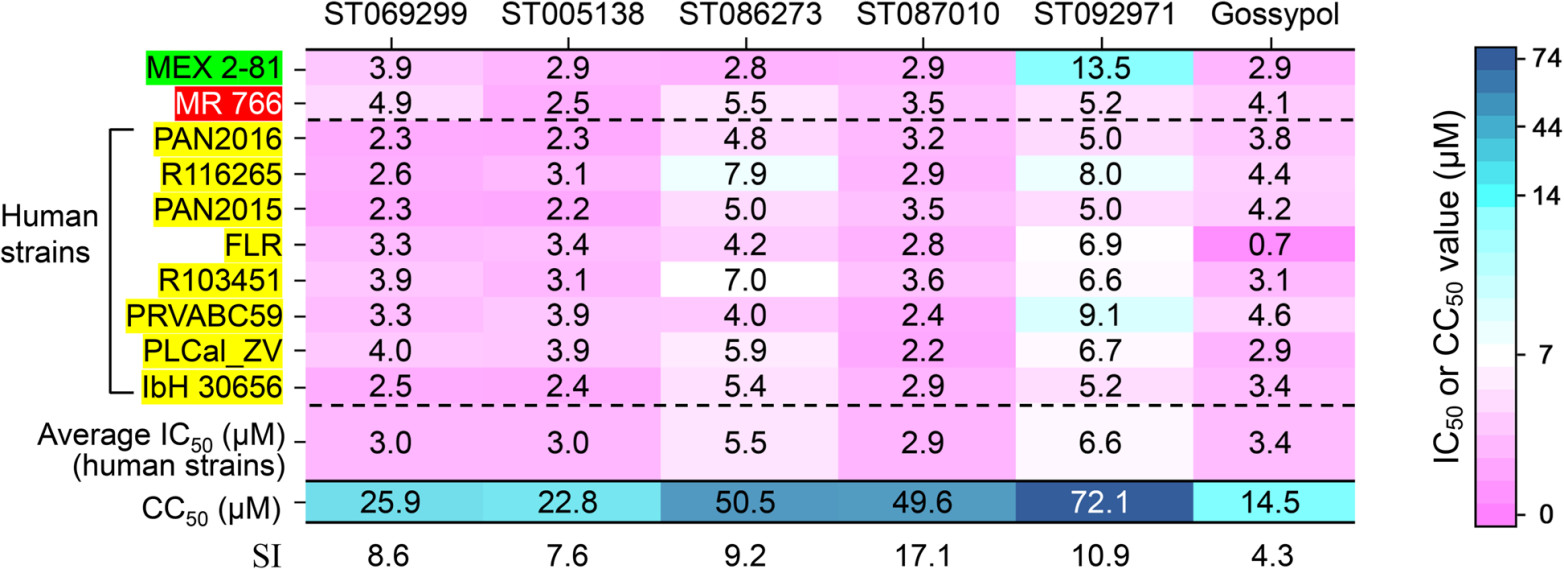
Heatmap of the IC_50_ values of gossypol derivatives against infection of ZIKV with different strains. Inhibitory activity of “hit” gossypol derivatives against ZIKV infection is expressed as IC_50_. Gossypol was used as a control. Human strains are highlighted in yellow, mosquito strain is in green, and rhesus strain is in red. The SI values were calculated based on the average IC_50_ values (human strains) and the respective CC_50_ values (i.e., CC_50_/IC_50_). The data are presented as the mean of duplicate wells in each experiment. The experiments were repeated twice with similar results. Magenta indicates a low IC_50_ or CC_50_ value whereas dark blue indicates a high IC_50_ or CC_50_ value

We then evaluated the broad-spectrum activity of these gossypol derivatives against nine other ZIKV strains from different hosts, such as human strains R116265, PAN2015, FLR, R103451, PRVABC59, PLCal_ZV, and IbH 30656; mosquito strain MEX 2-81; and rhesus macaque strain MR 766, which were isolated from various countries or territories (Mexico, Panama, Colombia, Honduras, Thailand, Nigeria, Uganda, and Puerto Rico) at different time periods (1947, 1968, 2013, 2015, and 2016) [41], and included gossypol as control. The IC_50_ values of some promising compounds, such as Temoporfin, Lopinavir-ritonavir, Sofosbuvir, and Nitazoxanide, against ZIKV infection have been reported to be 1.1, 4.8, 9.6, and 15.9 μM, respectively [48–50]. Although all gossypol derivatives inhibited infections of the 10 ZIKV strains tested, with the IC_50_ values at micromolar level, which were equal to, or better than, the reported compounds mentioned above, ST087010 exhibited more potent inhibitory activity against 8 of the 10 ZIKV strains tested, but slightly less potent than gossypol against ZIKV FLR and R103451 strains (Fig. 2). These data suggest the efficacious broad-spectrum activity of gossypol derivative ST087010 against multiple strains of ZIKV from different hosts, time periods, and countries/territories.

The selectivity index (SI) was used to evaluate the pharmaceutical safety of these derivatives. SI is defined as the ratio of the CC_50_ to the IC_50_. In general, the larger the SI value, the higher the safety of drugs. As shown in Fig. 2, the average IC_50_ values of the 8 human ZIKV strains (PAN2016, R116265, PAN2015, FLR, R103451, PRVABC59, PLCal_ZV, and IbH 30656) of “hit” gossypol derivatives ST069299, ST005138, ST086273, ST087010, and ST092971 and gossypol control were 3.0, 3.0, 5.5, 2.9, 6.6, and 3.4 μM, respectively. Thus, the SI values for these 6 compounds, which were calculated based on the average IC_50_ values (human strains) and the respective CC_50_ values, were 8.6, 7.6, 9.2, 17.1, 10.9, and 4.3, respectively (Fig. 2). Compared to gossypol, derivative ST087010 had reduced cytotoxicity in Vero E6 cells, and its CC_50_ was about 3.5-fold higher than that of gossypol; also, the SI value of ST087010 was much better than that of the other four derivatives which exhibited reduced anti-ZIKV activity (Figs. 1A and 2). Therefore, ST087010 was identified as the target gossypol derivative for further studies.

### Gossypol derivative ST087010 inhibited ZIKV infection by targeting the virus

**Fig. 3:**
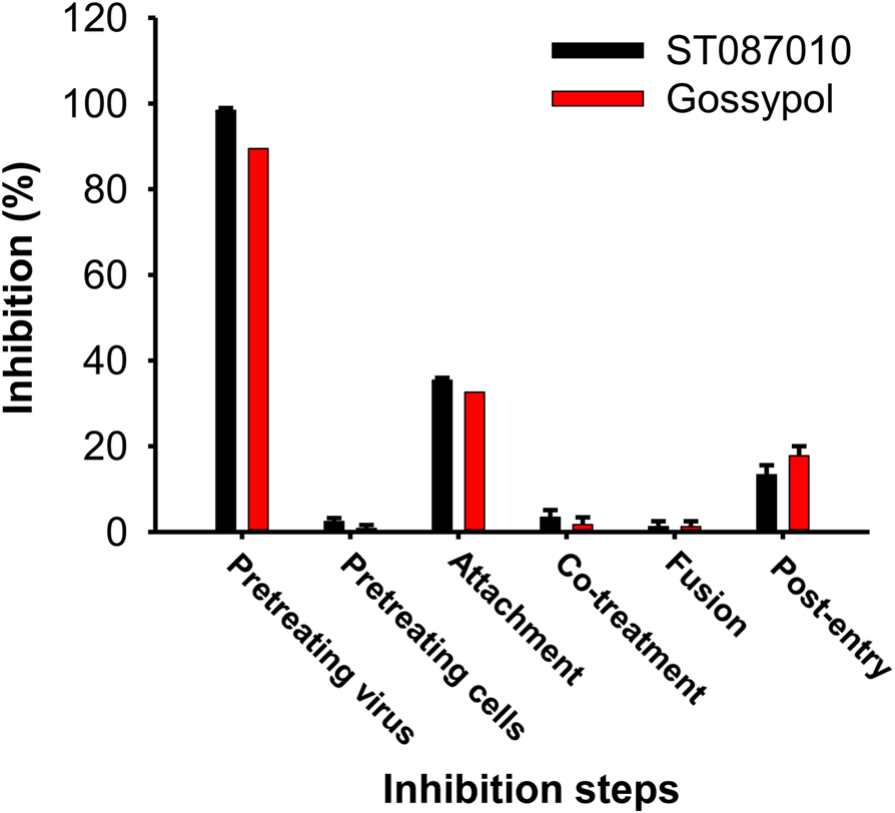
Potential inhibitory mechanism of gossypol derivative ST087010 against ZIKV infection. Time-of-addition experiment was performed in Vero E6 cells, and six specific conditions are shown as follows. (1) Condition 1: pretreatment of the virus. (2) Condition 2: pretreatment of cells. (3) Condition 3: attachment. (4) Condition 4: cotreatment. (5) Condition 5: fusion. (6) Condition 6: post-entry. The data are expressed as the mean % inhibition ± s.e.m. of duplicate wells. The experiments were repeated three times with similar results

To elucidate the potential inhibitory mechanism of gossypol derivative ST087010 in preventing ZIKV infection, we performed a time-of-addition assay to identify which step of the ZIKV life cycle may be affected. The results showed that ZIKV infection was almost completely inhibited after incubation of the virus with ST087010 at 37°C for 1 h, prior to incubating it with Vero E6 cells (condition 1). In contrast, <40% or <20% of ZIKV infection was inhibited, respectively, at viral attachment (condition 3) and post-entry (condition 6) steps, whereas no, or little, ZIKV infection was blocked in other conditions, such as pretreatment of cells (condition 2), cotreatment (condition 4), or fusion (condition 5) (Fig. 3), which are responsible for virus-cell binding, viral entry, and virus-cell membrane fusion, respectively [41]. These data suggest that derivative ST087010 inhibited ZIKV infection by mainly targeting the virus, thus preventing subsequent virus-cell binding, viral entry, or replication stages, a mechanism very similar to that of gossypol.

### Identification of binding region(s) of gossypol derivative ST087010 in ZIKV proteins

**Fig. 4:**
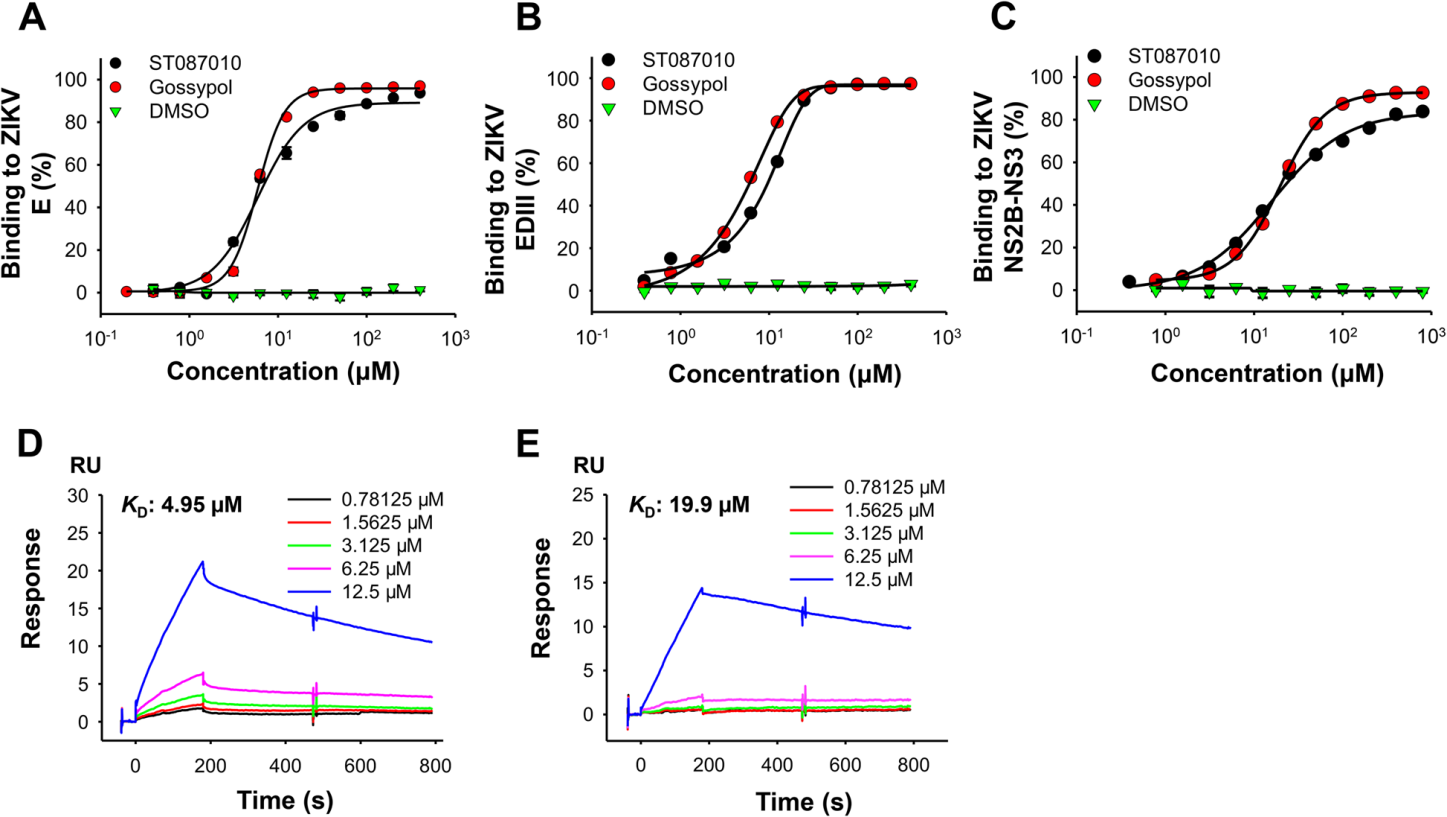
Binding affinity of gossypol derivative ST087010 to ZIKV proteins. Binding of ST087010 or gossypol control to ZIKV full-length envelope (E) protein (**A**), domain III of E (EDIII) protein (**B**), or NS2B-NS3 (**C**) protein was assessed by ELISA. The data are expressed as the mean ± s.e.m. of 4 wells, and DMSO was used as the negative control. Surface plasmon resonance (SPR) analysis of the binding between ST087010 and ZIKV EDIII (**D**) or NS2B-NS3 (**E**) protein. Binding affinity was shown as *K*_D_ (equilibrium dissociation constant). The experiments were repeated twice with similar results

In the life cycle of ZIKV, the E protein plays a key role in viral entry into target cells and subsequent fusion of virus and cell membranes, whereas NS2B and NS3 proteins consist of an important viral protease essential for post-entry/post-translational polyprotein processing, such as viral RNA replication, virion assembly, and virion release [49, 51]. Hence, similar to other flaviviruses, both ZIKV E and NS2B-NS3 protease serve as important therapeutic targets against ZIKV infection [24, 52–54]. Our previous data showed that gossypol binds to the ZIKV E protein (especially EDIII: E residues 298-409) [41], and the molecule docking analysis also indicated that gossypol binds to the ZIKV NS2B-NS3 protease (data not shown). Because of the structural similarity, we reason that ST087010 may also bind to these two ZIKV proteins. To identify the binding region(s) of gossypol derivative ST087010 in ZIKV E protein, we performed an ELISA by coating the plate with ZIKV full-length E or EDIII protein and tested for binding using ZIKV EDIII-specific mAb ZKA64-LALA. The binding between ST087010 and NS2B-NS3 proteins was performed by coating the ELISA plate with NS2B-NS3 proteins, followed by detection of binding, using NS2B-NS3 protein-immunized mouse sera. Results showed that ST087010 bound strongly to full-length E, EDIII, and NS2B-NS3 proteins, with 50% effective concentration (EC_50_) values of 6.47, 6.13, and 21.85 μM, respectively, which were similar to those of gossypol (Fig. 4A–C). Nevertheless, no binding was assessed between DMSO control and any of these proteins (Fig. 4A–C). Further results from the surface plasmon resonance (SPR) assay revealed that ST087010 bound potently to ZIKV EDIII or NS2B-NS3 protein with *K*_*D*_ (binding affinity) values of 4.95 and 19.9 μM, respectively (Fig. 4D, E).

**Fig. 5:**
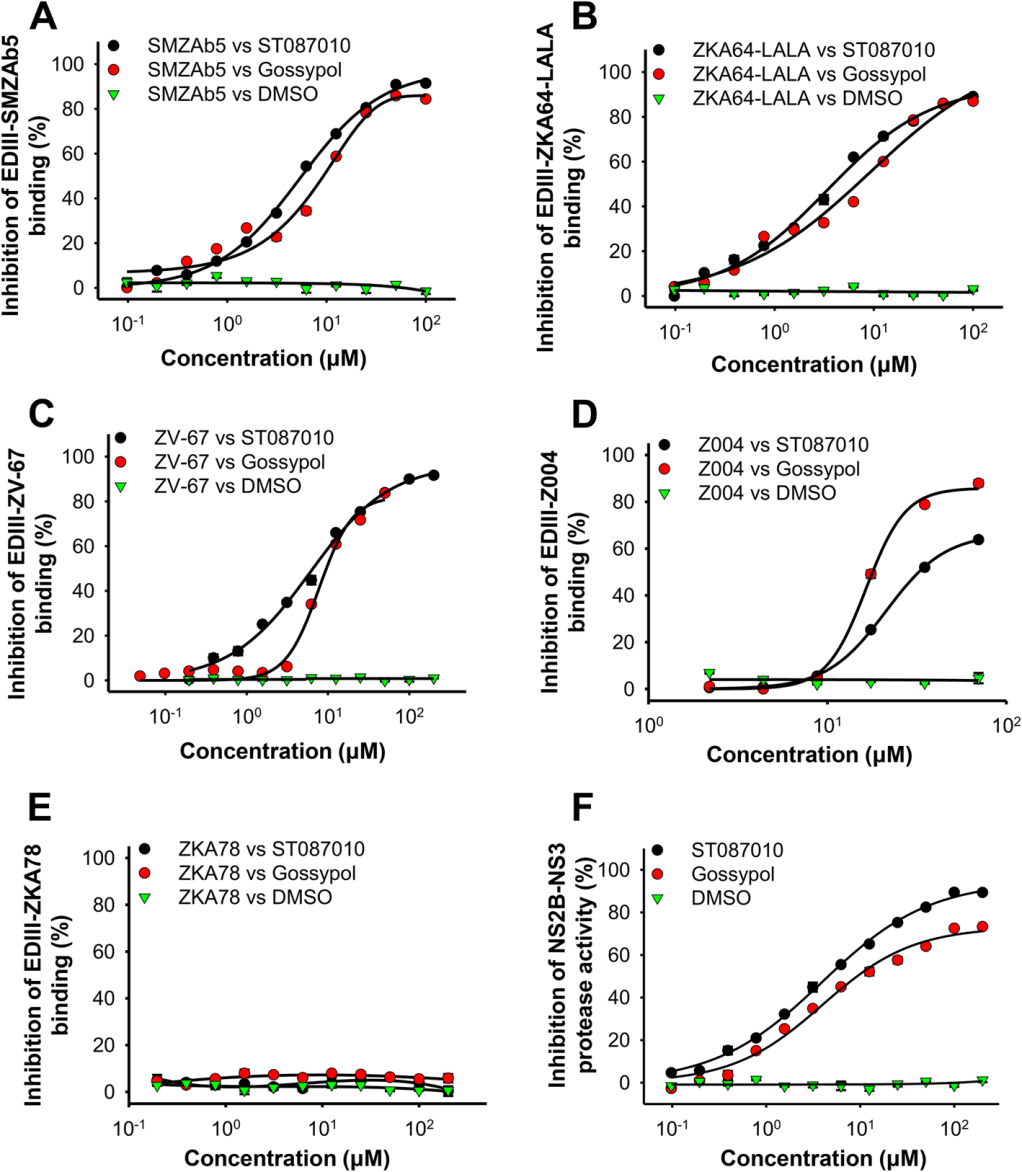
Ability of gossypol derivative ST087010 to inhibit binding of ZIKV EDIII to EDIII-specific neutralizing mAbs, as well as ZIKV NS2B-NS3 protease activity. **A**–**D** Percent inhibition (% inhibition) of EDIII-mAb binding was calculated in the presence or absence of serially diluted compounds (ST087010 or gossypol control) based on the ELISA result. Four ZIKV EDIII-specific mAbs were used for testing, and IC_50_ values were calculated. ZIKV EDI/DII-specific mAb ZKA78 (**E**) and DMSO were used as controls. **F** Ability of ST087010 in inhibition of ZIKV NS2B-NS3 protease activity. Percent inhibition (% inhibition) of protease activity was measured in the presence or absence of serially diluted compounds, and IC_50_ values were calculated. The data are expressed as the mean ± s.e.m of 4 wells. The experiments were repeated twice with similar results

We next identified the potential binding site(s) of ST087010 in the ZIKV EDIII region using an ELISA competition assay. As such, the plate was coated with ZIKV EDIII protein, and the binding of ZIKV EDIII to EDIII-specific neutralizing mAbs, including SMZAb5, ZKA64-LALA, ZV-67, and Z004, was assessed in the presence of ST087010 at serial dilutions. The results of gossypol in this experiment were consistent with our previous reports [41]. Similar to gossypol, the results indicated that ST087010 effectively blocked the binding between EDIII and these mAbs in a dose-dependent manner, resulting in the IC_50_ values of 5.72, 4.17, 6.70, and 44.51 μM, respectively, for SMZAb5, ZKA64-LALA, ZV-67, or Z004; in contrast, DMSO control had no ability to block the binding of EDIII to any of these mAbs (Fig. 5A–D). As expected, since ZIKV EDI/II-specific mAb control ZKA78 did not bind to EDIII protein, no inhibition was assessed by ST087010 or gossypol control (Fig. 5E). The above ZIKV EDIII-specific mAbs have been previously shown to recognize epitopes, including the lateral ridge, such as residues 309–314, 331–337, 368, 370, 371, and 393–397, of the ZIKV EDIII protein [55–57]. Therefore, consistent with the results on gossypol reported previously [41], ST087010 also potentially binds to these epitopes of ZIKV EDIII protein, thus blocking the binding between EDIII and EDIII-specific mAbs.

Since ST087010 bound to ZIKV NS2B-NS3 protein, we wanted to know if it could block the cleavage of this protease. To address this question, a fluorescence-based inhibition assay was performed in the presence of serially diluted ST087010 and measured for the fluorescence signals. The results demonstrated that ST087010 did inhibit ZIKV NS2B-NS3 protease cleavage in a dose-dependent manner with an IC_50_ value of 4.84 μM, whereas DMSO control had no inhibitory activity against this cleavage (Fig. 5F). These results confirmed that ST087010 has strong inhibitory activity against ZIKV NS2B-NS3 protease.

### Cytotoxicity and in vitro broad-spectrum inhibitory activity of gossypol derivative ST087010 against infection of DENV-1-4 strains

We further assessed the broad-spectrum activity of gossypol derivative ST087010 against infections of other flaviviruses, such as DENV, and compared the results with those of gossypol. As such, four serotypes of DENV human strains, including DENV-1-V1792, DENV-2-V594, DENV-3-V1043, and DENV-4-PR-06-65-740, were tested by plaque assay for inhibition of viral infection in LLC-MK2 cells. Although ST087010 was slightly less potent than gossypol against infections of DENV-1, DENV-2, and DENV-4 strains tested, its cytotoxicity was much lower than that of gossypol, with a CC_50_ value of 47.2 μM (Table 1). By comparing inhibitory activity and cytotoxicity profiles, we found that ST087010 had SI values of 15.7, 14.8, 14.2, and 15.8, respectively, against infections of DENV-1, DENV-2, DENV-3, and DENV-4, which were higher than those of gossypol (Table 1). These data suggest strong broad-spectrum activity of ST087010 against DENV-1-4 infections in vitro with lower cytotoxicity and higher safety, as compared to gossypol.

**Table 1:** Cytotoxicity and in vitro inhibitory activity of gossypol derivative ST087010 against infections of DENV-1-4 strains^a^

Gossypol derivative	CC_50_ (μM)	IC_50_ (μM)							
		DENV-1-V1792	SI	DENV-2-V594	SI	DENV-3-V1043	SI	DENV-4-PR-06-65-740	SI
ST087010	47.20 ± 1.35	3.01 ± 0.11	15.7	3.19 ± 0.01	14.8	3.33 ± 0.08	14.2	2.98 ± 0.18	15.8
Gossypol	19.06 ± 1.09	2.06 ± 0.13	9.3	1.91 ± 0.01	10.0	3.42 ± 0.11	5.6	2.83 ± 0.07	6.7

^a^The cytotoxicity and inhibitory activity of gossypol derivative ST087010 were assessed in LLC-MK2 cells, and gossypol was included as a control. Cytotoxicity is expressed as CC_50_. Inhibitory activity against DENV-1-4 infections is expressed as IC_50_. Selectivity index (SI) was calculated based on the values of CC_50_/IC_50_. The data are presented as the mean ± standard error of the mean (s.e.m.) of duplicate wells. The experiments were repeated twice with similar results

Although there are some variations in the amino acid sequences of E and NS2B-NS3 proteins of ZIKV and DENV strains tested in this study (Additional file 1: Fig. S1) [41], here we found that gossypol derivative ST087010 effectively inhibited the infections of all these virus strains. Our data demonstrated the broad-spectrum activity of this compound against ZIKV and DENV-1-4 infections.

### Gossypol derivative ST087010 protected adult *Ifnar1*^*−/−*^ mice from lethal ZIKV challenge and inhibited viral infection

**Fig. 6:**
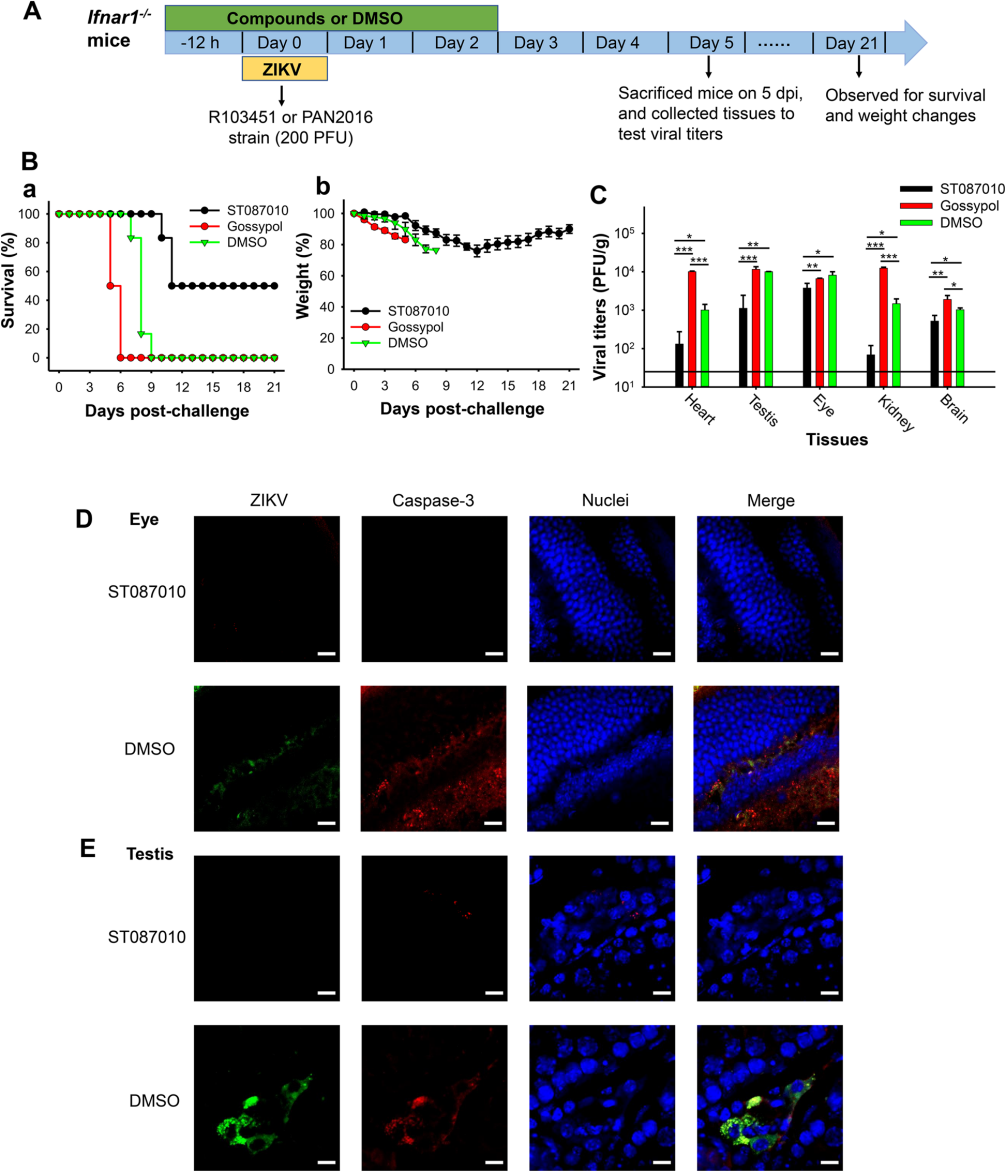
Efficacy of gossypol derivative ST087010 in protecting *Ifnar1*^*−/−*^ mice from lethal ZIKV challenge. **A** Schematic diagram of experimental procedures. Mice were intraperitoneally (i.p.) treated with ST087010, gossypol (control), or DMSO (negative control), as shown in the figure. **B** The treated mice were infected with ZIKV human strain R103451 (200 PFU/mouse), followed by evaluation of survival rate (a) or weight changes (b) for 21 days. The data are expressed as the mean ± s.e.m. of 6 mice in each group. In a separate experiment, ST087010 or gossypol-treated mice were infected with ZIKV human strain PAN2016 (200 PFU/mouse). Five days post-infection (dpi), viral titers were assessed in tissues by plaque assay (**C**), and ZIKV or caspase-3 signals were measured in the eye (**D**) and testis (**E**) tissues by immunofluorescence staining. The data (**C**) are expressed as the mean ± s.e.m. of 5 mice in each group, and significant differences among mice treated with ST087010, gossypol, and DMSO in the respective tissues (heart, testis, eye, kidney, or brain) were compared and are shown as *, **, and ***. The detection limit is 25 PFU/g. ZIKV (green) and caspase-3 (red) (in **D**, **E**) were stained with anti-ZIKV and anti-active caspase-3 antibodies, respectively. Nuclei (blue) were stained with DAPI (4′,6-diamidino-2-phenylindole). Representative images of immunofluorescence staining are shown. Magnification: 63×, and scale bar: 10 μm

The above in vitro data identified improved anti-ZIKV activity of gossypol derivate ST087010. These in vitro experimental results revealed that it is critical for ST087010 to be interacted with the virus before the virus reaches a cell, suggesting that ST087010 is suitable for the prevention and treatment of ZIKV infection. Thus, we tested its in vivo antiviral effect in adult *Ifnar1*^*−/−*^ mice, which are highly susceptible to ZIKV infection. We selected several recent ZIKV strains (R103451, PAN2016, and R116265) isolated from humans in order to verify the broad-spectrum inhibitory activity of ST087010. Mice were intraperitoneally (i.p.) treated with ST087010 (20 mg/kg), gossypol (20 mg/kg), or DMSO control 12 h before infection and 6, 24, and 48 h post-infection, and they were i.p. infected with either ZIKV human strain R103451, followed by observation of survival and weight changes for 21 days, or ZIKV human strain PAN2016, followed by detection of ZIKV titers in different tissues at 5 days post-infection (dpi) (Fig. 6A). The 5 dpi was selected because it was most suitable for detecting viral titers in the tissues of challenged mice in our preliminary experiments. ZIKV may not enter the tissue if it is tested too early, or ZIKV titers in the tissues may be hard to be detected after 5 dpi.

As shown in Fig. 6Ba, all mice treated with DMSO died at 9 dpi, whereas treatment with ST087010 protected 50% of the mice from death caused by ZIKV infection. In contrast, no mice treated with gossypol survived at 6 dpi, and these mice even died earlier than DMSO-treated mice (Fig. 6Ba), suggesting that the death in the gossypol-treated mice might result from the toxicity of gossypol. In addition, gossypol or DMSO-treated mice showed increased weight loss, whereas the mice treated with ST087010 presented weight loss (less than 25%) during 6–12 dpi without severe symptoms, steadily increasing in weight to 85% of the body weight before the virus infection until 21 dpi (Fig. 6Bb). Moreover, ST087010-treated mice had significantly reduced viral titers in the heart, testis, eye, kidney, and brain compared to gossypol or DMSO-treated mice. However, gossypol-treated mice even had significantly higher viral titers in the heart, kidney, and brain than DMSO-treated mice (Fig. 6C), suggesting, again, that such consequence might have been potentially caused by the toxicity of gossypol. The important toxic effect of gossypol is its interference with immune function, reducing animal’s resistance to infection and leading to increased virus replication [58]. These data indicate strong activity of gossypol derivative ST087010 in protecting mice against ZIKV-caused death and weight loss and preventing viral replication in challenged mice from two different human strains tested.

To identify potential mechanisms of ST087010 in inhibiting viral infection and ZIKV-caused tissue damage, we stained eye and testis tissues from ST087010- or DMSO control-treated mice collected at 5 dpi for an activated form of caspase-3, an apoptotic marker. The results from immunofluorescence staining indicated that undetectable, or diminished, staining for caspase-3 was observed in the eye and testis tissues from mice treated with ST087010 as compared to the mice treated with DMSO, which was accompanied by undetectable straining of ZIKV^+^ signals (Fig. 6D, E). These data suggest that gossypol derivative ST087010 prevented ZIKV-caused apoptosis and cell death.

### Gossypol derivative ST087010 was safe for pregnant *Ifnar1*^*−/−*^ mice and their fetuses and pups

**Fig. 7:**
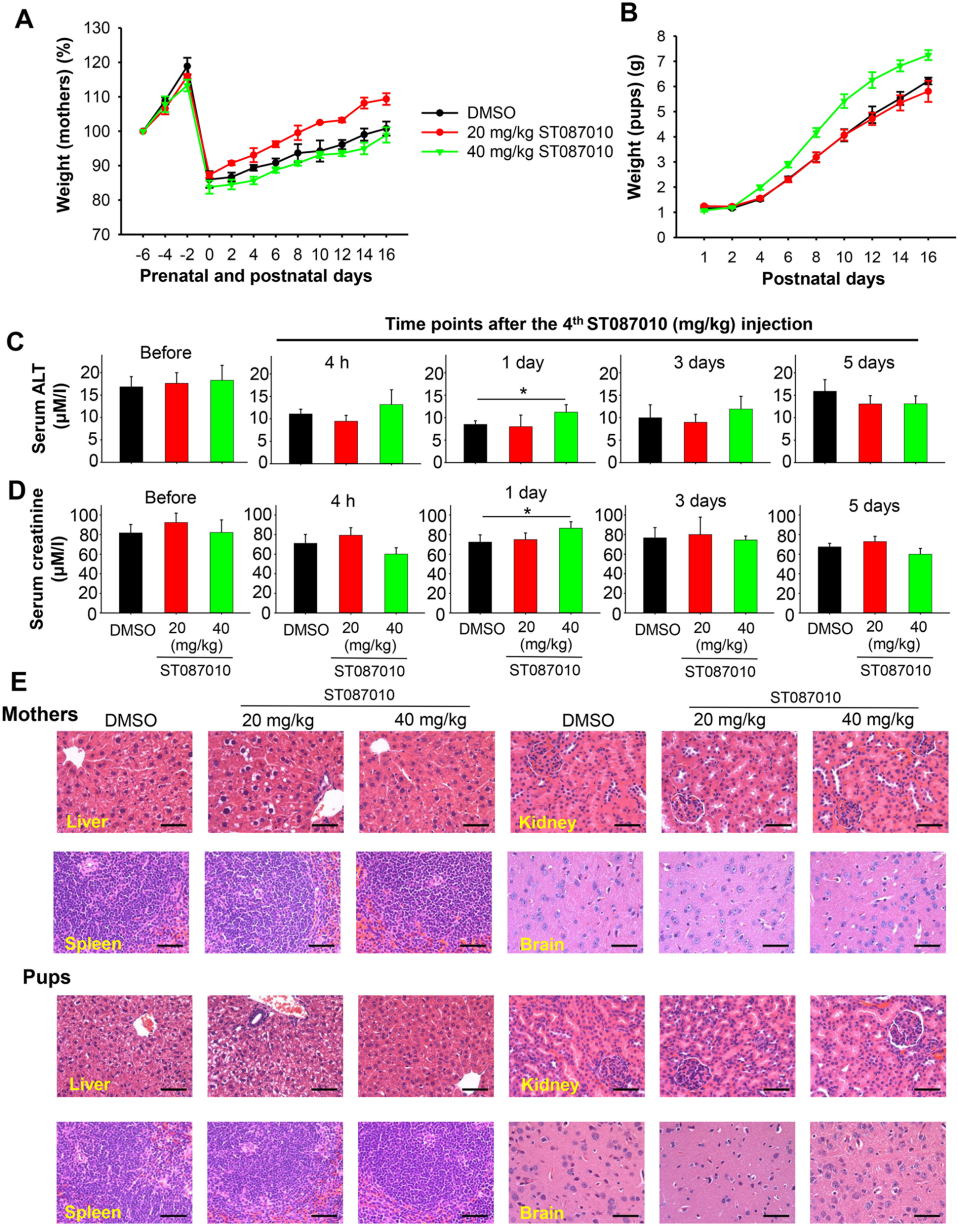
Safety profile of gossypol derivative ST087010 in pregnant *Ifnar1*^*−/−*^ mice and their pups. **A** Weight changes of pregnant mothers at prenatal and postnatal time points. **B** Weight changes of pups at different postnatal time points. Alanine transaminase (ALT) (**C**) and creatinine (**D**) levels in sera of pregnant mice were measured by ALT assay and creatinine assay, respectively, before and 4 h, 1, 3, and 5 days post-last injection of ST087010 or DMSO control. The data (in **A**–**D**) are expressed as the mean ± s.e.m. of 5 mice in each group, and significant differences (*) between ST087010 (40 mg/kg) and DMSO groups at 1 day post-treatment are shown (in **C**, **D**). **E** Hematoxylin and eosin (H&E) staining of tissues, including the liver, spleen, kidney, and brain, from ST087010 or DMSO-treated mothers and their pups. Scale bar, 50 μm

It is important that ZIKV therapeutics have strong safety to pregnant individuals since the virus causes congenital ZIKV syndrome with significant growth abnormalities to fetuses. Here, we studied the safety of ST087010 in pregnant *Ifnar1*^***−****/****−***^ mice, as previously described. The results showed that pregnant mice (Fig. 7A) and their pups (Fig. 7B) had similar weight, or slight weight changes, after the mice received ST087010 at 20 or 40 mg/kg, or DMSO control, suggesting that ST087010 did not result in significant damage to the pregnant mice and their fetuses, although weight loss is not the only effect of the damage, and thus pups grew normally.

We also measured alanine transaminase (ALT) (Fig. 7C) and creatinine (Fig. 7D) levels in the sera of mice prior to, and after, receiving ST087010 or DMSO at different time points. Although ALT and creatinine are not the only measurements that determine the function of the liver and kidney, they can reflect changes in liver and kidney function to a certain extent. The results showed that no significant differences of ALT and creatinine levels were observed in the mice before injection and at 4 h and 3 or 5 days after the last injection of ST087010, suggesting that injection of pregnant mice with ST087010 at 20 or 40 mg/kg did not change their hepatic and renal function at these time points. In addition, serum ALT and creatinine levels were significantly different between high-dose ST087010-treated mice (40 mg/kg) and DMSO-treated mice at 1 day after injection, whereas no significant difference was noted between the two groups after mice received a low dose of ST087010 (20 mg/kg), a dose which still showed strong anti-ZIKV activity in vivo. These data suggest that ST087010 at a high dose (40 mg/kg) might have some slight effect on the hepatic and renal function of pregnant mice, whereas it will not affect the function of the liver and kidney when it is used at a low lose (20 mg/kg) but still with inhibitory activity.

Histopathological analysis of 4′,6-diamidino-2-phenylindole (DAPI)-stained tissues from mothers and pups indicated that the liver, spleen, kidney, and brain of mice treated with ST087010 at 20 or 40 mg/kg did not present pathological changes, as compared to those of mice treated with DMSO (Fig. 7E). In addition, no inflammation or cell infiltration was observed in the mice treated with ST087010 at both low and high doses (Fig. 7E), suggesting that gossypol derivative ST087010 can be considered safe for pregnant mice and their fetuses, even at the high dose of 40 mg/kg.

### Gossypol derivative ST087010 blocked vertical transmission of ZIKV in pregnant *Ifnar1*^*−/−*^ mice, preventing fetal death

ZIKV may vertically transmit from mothers to fetuses, causing fetal damage or death. We tested the efficacy of ST087010 in blocking ZIKV vertical transmission in *Ifnar1*^*−/−*^ mice. Pregnant mice (embryonic day (E)12–14) were i.p. injected with ST087010 (20 mg/kg) or DMSO (control) 12 h before infection and 6, 24, and 48 h post-infection, and they were i.p. infected with a ZIKV human strain (R116265, 10^3^ plaque-forming unit: PFU) as previously described [59, 60]. This was followed by a collection of sera and tissues at 5 dpi, detection of viral titers by plaque assay in the sera, placenta, fetal brain, and amniotic fluid, as well as observation of morphological changes in uteri and fetuses, and apoptosis in placentas.

**Fig. 8:**
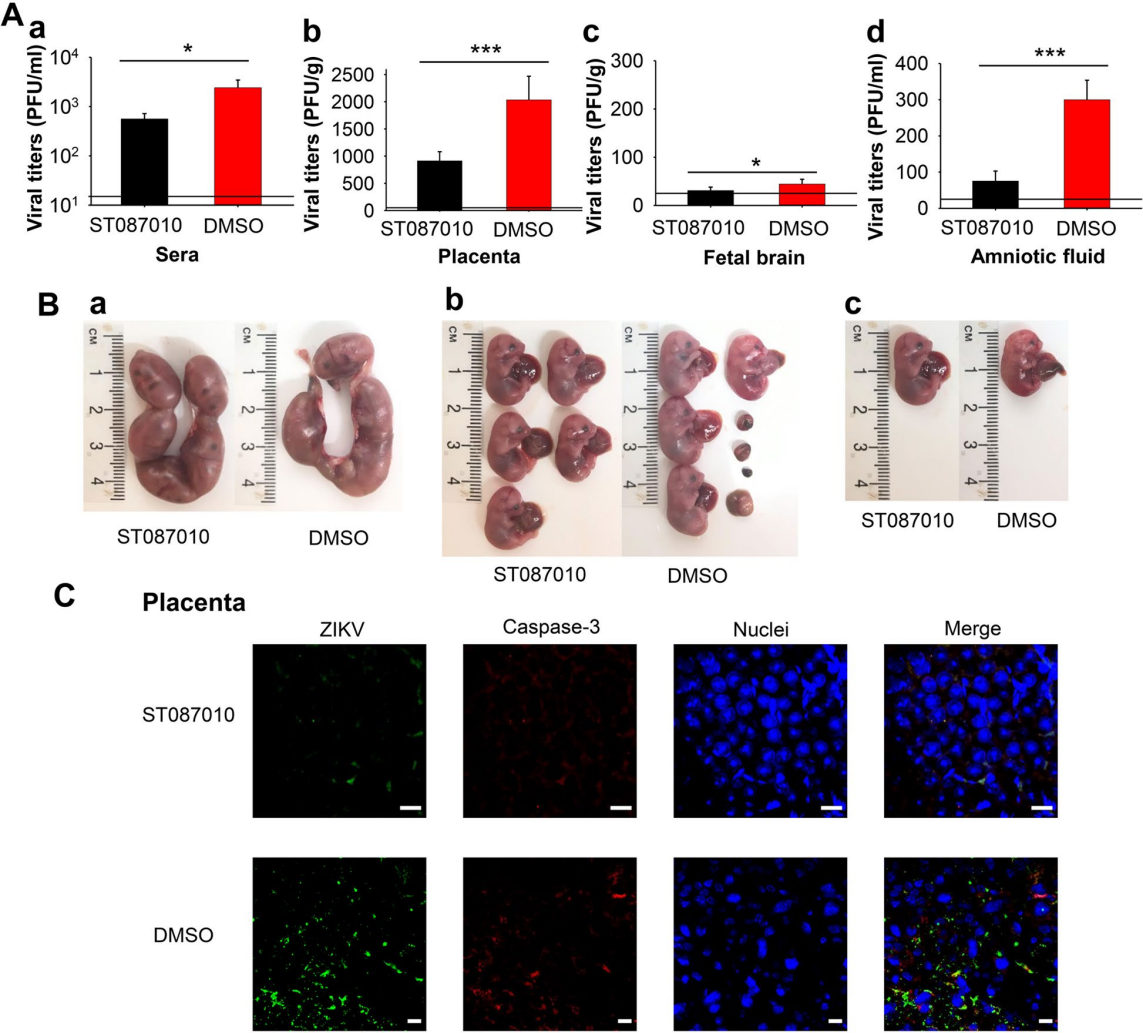
Efficacy of gossypol derivative ST087010 in protecting pregnant *Ifnar1*^*−/−*^ mice and their fetuses against ZIKV challenge. Pregnant mice were i.p. treated with ST087010 or DMSO (control) and infected with ZIKV human strain R116265 (10^3^ PFU/mouse), as described in the “Methods” section. **A** Detection of viral titers by plaque assay in the sera (a), placenta (b), fetal brain (c), and amniotic fluid (d) at 5 dpi. The data are expressed as the mean ± s.e.m. of 5 mice in each group, and significant differences between ST087010 and DMSO groups in the sera, placenta, fetal brain, and amniotic fluid, respectively, are shown. The detection limit is 25 PFU/ml (for sera and amniotic fluid) or 25 PFU/g (for the placenta and fetal brain). **B** Representative images of the morphology of mouse uteri and fetuses at 5 dpi are shown. (a) E17-19 uteri from pregnant mice challenged at E12-14. Fetal morphology (b) and fetal size (c). **C** ZIKV or caspase-3 signals were measured in the placenta by immunofluorescence staining. ZIKV (green) and caspase-3 (red) were stained with anti-ZIKV and anti-active caspase-3 antibodies, respectively. Nuclei (blue) were stained with DAPI. Representative images of immunofluorescence staining are shown. Magnification, 63×, and scale bar 10 μm

The results showed that viral titers were significantly reduced in ST087010-treated mouse sera (Fig. 8Aa), placenta (Fig. 8Ab), fetal brain (Fig. 8Ac), and amniotic fluid (Fig. 8Ad) when compared to those of control mice treated with DMSO. In addition, part of the fetuses from DMSO-treated pregnant mice died in uteri, while the fetuses from ST087010-treated mice were all in good condition, and their uteri had intact morphology (Fig. 8Ba-b). Particularly, as shown in Fig. 8Bc, the size of fetuses treated with DMSO was smaller than that of the fetuses treated with ST087010, suggesting growth restriction. These data demonstrate that ST087010 prevented ZIKV-infected pregnant mice from vertical transmission and resultant ZIKV-caused fetal growth restriction and fetal death.

The results from immunofluorescence staining indicated undetectable, or diminished, staining of ZIKV^+^, or caspase-3, signals in the placental tissues from mice treated with ST087010, as compared to those from DMSO-treated mice (Fig. 8C), suggesting that ST087010 prevented ZIKV-associated apoptosis and viral replication.

### Gossypol derivative ST087010 inhibited DENV-2 replication in *Ifnar1*^*−/−*^ mice

**Fig. 9:**
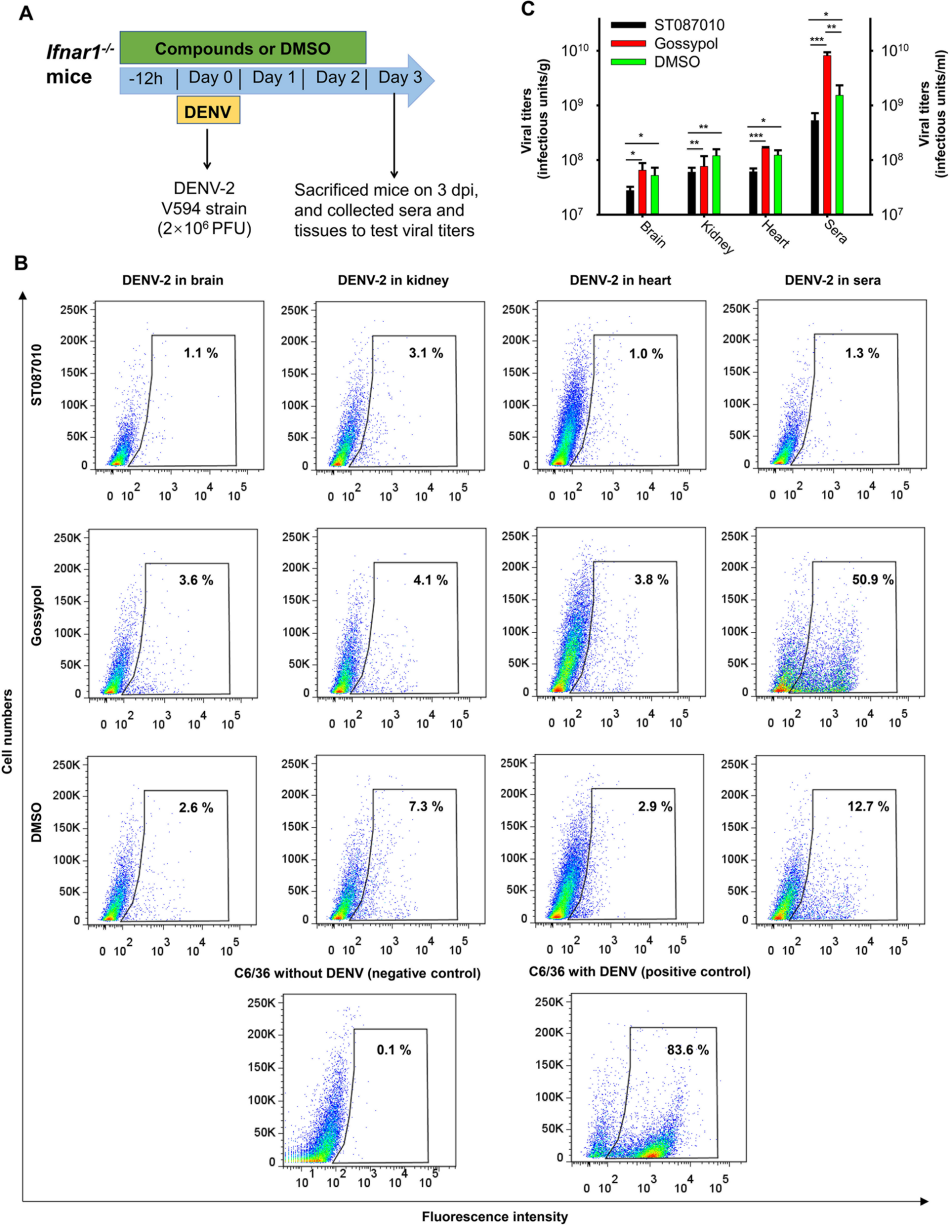
Efficacy of gossypol derivative ST087010 in protecting *Ifnar1*^*−/−*^ mice from DENV-2 challenge. **A** Schematic diagram of experimental procedures. Mice were i.p. treated with ST087010, gossypol control, or DMSO (negative control) and infected with DENV-2 human strain V594. **B** Representative images of DENV-2 titers in the brain, kidney, heart, and sera analyzed by flow cytometry at 3 dpi. C6/36 cells with or without DENV-2 infection were used as positive and negative controls, respectively. The percentage of infected cells to the total number of cells is shown in each figure. **C** Detection of DENV-2 titers in challenged mouse tissues and sera at 3 dpi. Viral titers are expressed as infectious units/g (for the brain, kidney, or heart) or infectious units/ml (for the sera), as assessed by flow cytometry assay (see the “Methods” section) and calculated based on **B**. Viral titers (infectious units/ml or infectious units/g) were calculated as the formula ((% of infected cells)×(total number of cells)×(dilution factor)/(amount of inoculum added to cells)). Differences among mice treated with ST087010, gossypol, or DMSO in the respective tissues (brain, kidney, and heart) or sera were compared. *, **, and *** indicate significant differences of DENV-2 infection between ST087010 and gossypol or DMSO groups, or between gossypol and DMSO groups. The data are presented as the mean ± s.e.m of 5 mice in each group. The experiments were repeated twice with similar results

To detect the inhibitory ability of ST087010 in preventing DENV infection in vivo, 3- to 4-week-old *Ifnar1*^*−/−*^ mice were i.p. injected with ST087010 (20 mg/kg), gossypol (20 mg/kg), or DMSO 12 h before and 6, 24, and 48 h after infection. The mice were i.p. infected with DENV-2 (human strain V594, 2×10^6^ PFU) and measured for viral loads (based on the number of virus-infected cells) in tissues (brain, kidney, and heart) and sera collected at 3 dpi (Fig. 9A). Since *Ifnar1*^*−/−*^ mice are more susceptible to DENV-2 than to other serotypes of DENV, we thus used DENV-2 at a high dose to infect these mice to ensure that the virus can be detected in the tissues of DMSO-treated control mice, and selected 3 dpi as an appropriate date for the detection of viral titers in the tissues of challenged mice. C6/36 cells were infected with supernatants of tissue samples and sera, and the number of infected cells was determined by a flow cytometry-based assay. The results revealed that mice treated with ST087010 had significantly reduced numbers of infected cells (viral titers) in the brain, kidney, heart, and sera, as compared to those treated with gossypol and DMSO (Fig. 9B, C), suggesting inhibition of DENV replication in the ST087010-treated mice. These data demonstrate broad-spectrum in vivo inhibitory activity of gossypol derivative ST087010 against other flaviviruses, such as DENV.

## Discussion

Development of safe and efficacious therapeutics is critical to stop ZIKV infection and control its adverse effects, particularly in pregnant individuals. Although early results towards a ZIKV vaccine are promising [61, 62], the cross-reactivity of ZIKV and DENV antibodies has been shown to enhance infection and disease in cell culture and murine models [63, 64]. Due to the potential challenges this phenomenon may present to the safety and efficacy of vaccines, small-molecule drugs will provide a complementary strategy to limit ZIKV viral burden, viral persistence, and disease severity without the risk of cross-reactive antibodies. However, the antiviral activity of small-molecule inhibitors, such as gossypol [41], needs to be improved, and their toxicity needs to be reduced, particularly in pregnant women and newborns infected with ZIKV. Similar to ZIKV, DENV-1-4 have overlapping epidemic areas and similar seasonal correlations; coinfection of these flaviviruses has been increasingly seen as a challenge [65]. Therefore, development of broad-spectrum antiviral therapeutics targeting both viruses is especially important in preventing their coinfections, in addition to treating respective diseases caused by both viruses.

We previously identified gossypol as an effective agent to inhibit ZIKV and DENV infections in vitro [41]. Nevertheless, gossypol has high cytotoxicity [66–68], and, thus, it is not suitable for treating ZIKV and DENV infections in vivo, particularly for pregnant women and their fetuses and babies infected with ZIKV. Therefore, elucidation of gossypol derivatives with less cytotoxicity but improved inhibitory activity will be critical to treat both flaviviruses. After screening a series of gossypol derivative compounds for their cytotoxicity and in vitro antiviral activity against ZIKV infection, we found that ST087010 had significantly reduced cytotoxicity compared to that of gossypol. Particularly, it exhibited potent and broad-spectrum inhibitory activity against at least 10 ZIKV strains. Moreover, ST087010 also potently inhibited infections of DENV-1-4 serotypes in vitro with much lower cytotoxicity than gossypol. Therefore, this gossypol derivative was further evaluated for its in vivo inhibitory activity against ZIKV and DENV infections and assessed for its in vivo toxicity in pregnant mice and their fetuses/pups.

The *Ifnar1*^***−****/****−***^ mice are susceptible to ZIKV infection, which is lethal and presents much more severe disease symptoms than natural infection [69–72], providing a sensitive in vivo model for identifying effective antiviral treatments. Accordingly, this mouse model was used to evaluate the protective efficacy of gossypol derivative ST087010. As expected, the ST087010 derivative extended the time of survival and reduced the mortality of *Ifnar1*^***−****/****−***^ mice infected with ZIKV, coinciding with the decreased virus titers in different tissues, including the brain and testis, two key organs with severe outcomes after ZIKV infection [12, 73]. Moreover, ST087010 also protected DENV-2-challenged *Ifnar1*^***−****/****−***^ mice against viral replication in sera and tissues, such as the brain, kidney, and heart. Currently, a number of drugs, such as emricasan, niclosamide, and inhibitors of cyclin-dependent kinases, including those approved by the FDA for human use against other viral pathogens [74], inhibit ZIKV and DENV replication in vitro. However, their efficacy in preventing ZIKV and/or DENV infections in vivo has not been investigated [74–76]. Here, we confirm the broad-spectrum activity of gossypol derivative ST087010 in protecting against both ZIKV and DENV infections in vivo.

ZIKV may transmit vertically with severe fetal demise [2, 77]. Thus, effective treatments should reduce viral replication in pregnant women, minimizing the risk of vertical transmission to the fetus [78]. Although compounds, such as Novobiocin and 7-deaza-2′-C-methyladenosine, have been tested against ZIKV infection in mouse models [49, 79], their protective efficacy against ZIKV vertical transmission has not been evaluated. Pregnant mice, such as *Ifnar1*^***−****/****−***^, A129, and AG129 mice, infected with ZIKV may present maternal infection and neonatal microcephaly, leading to fetal and/or pup demise. As such, they are considered appropriate models for investigating ZIKV transmission from the mother to the fetus [72, 77, 80, 81]. Treatment of pregnant A129 mice with compound NITD008 or treatment of pregnant SJL mice with compound Sofosbuvir reduced viral loads in the placenta and fetal brain, respectively, thereby preventing vertical transmission of ZIKV to the fetus [78, 82]. Here we demonstrated that compound ST087010 potently blocked ZIKV vertical transmission in pregnant *Ifnar1*^***−****/****−***^ mice, preventing ZIKV-caused fetal death. Particularly, ST087010 was safe for pregnant *Ifnar1*^***−****/****−***^ mice and their fetuses and pups. As a result, gossypol derivative ST087010 exhibited anti-ZIKV activity in pregnant mice; it also inhibited DENV-2 infection in vivo. Future work will be needed to confirm the metabolism of gossypol derivative ST087010 in vivo and its ability to cross the placental barrier and/or blood-brain barrier.

## Conclusions

In summary, this study identified gossypol derivative ST087010 for its strong potency against ZIKV and DENV infections in vitro and in vivo, but with low toxicity for good safety. These findings indicate the possibility for further development of this small-molecule compound as a novel and promising antiviral drug to treat ZIKV or DENV infection in high-risk populations, particularly pregnant women. The strong safety profile and high potency of this compound also determine its role for possible use clinically to reduce the risk of maternal-fetal transmission of ZIKV, as well as to inhibit DENV infection. This study also suggests the potential to further develop ST087010 into an effective broad-spectrum anti-flavivirus drug.

## Methods

### Mice

Adult male (3–4 or 7–8-week-old) and pregnant female (8–12-week-old, E12-14) *Ifnar1*^**−***/***−**^ mice were used in the study. The mice were placed in appropriate ventilated cages (34.3 cm × 29.2 cm × 15.5 cm, L × W × H; up to four adult mice per cage, and one female/one male per cage for breeding pairs) with 1/8-inch Bed-o-Cob bedding, and given commercial rodent food (5P76) and acidified water. The mice were provided with a 12-h light/12-h dark cycle, enrichment devices (including PVC tunnels and Nylabones/Gumabones), and nesting materials (Nestlets, Enviro-Dri, and Glatfelter Tea Bag filter paper).

### Cells and viruses

Vero E6 (African green monkey kidney, ATCC CRL-1586) and LLC-MK2 (rhesus monkey kidney, ATCC CCL 7.1) cells were cultured in Dulbecco’s modified Eagle’s medium (DMEM) containing 8% FBS and 1% penicillin and streptomycin (P/S) to maintain cell growth and prevent potential contamination. C6/36 cells (*Aedes albopictus* mosquito, ATCC CRL-1660) were cultured in Eagle’s Minimal Essential Medium (EMEM) containing 6% FBS and 1% P/S as described above. The cells were tested for mycoplasma monthly using Mycoplasma Detection Kit (ATCC), inspected daily for sterility, morphology, and adherence, and maintained for about 8–10 passages. Once cells were identified for contamination and sterility problems or morphology changes, they were immediately replaced with a new frozen stock or a newly purchased vial. ZIKV strains isolated from humans, including PAN2015 (2015/Panama), PAN2016 (2016/Panama), R116265 (2016/Mexico), FLR (2015/Colombia), R103451 (2015/Honduras), PRVABC59 (2015/Puerto Rico), PLCal_ZV (2013/Thailand), and IbH 30656 (1968/Nigeria); from mosquitos, including MEX 2-81 (2016/Mexico); and from rhesus macaques, including MR 766 (1947/Uganda), were used. Four serotypes of DENV human isolates, including DENV-1-V1792 (2007/Vietnam), DENV-2-V594 (2006/Puerto Rico), DENV-3-V1043 (2006/Puerto Rico), and DENV-4-PR-06-65-740 (2006/Puerto Rico), were used. ZIKV and DENV-1-4 were maintained in Vero E6 and C6/36 cells, respectively, and their viral titers were assessed by plaque assay [83] in Vero E6 and LLC-MK2 cells, respectively. Vero E6 and C6/36 cells are laboratory-adapted cells that have been most frequently used to propagate ZIKV and DENV, respectively, and test antiviral activity [84–87]. The reasons for choosing Vero E6 and LLC-MK2 cells, instead of human-derived cells, are that Vero E6 cells are the gold standard for plaque assay to determine the titer of ZIKV stocks, infected cell culture supernatants, and animal tissue homogenates, and plaque assay of DENV has been performed frequently using LLC-MK2 cells [84, 87, 88]. C6/36 cells can be easily infected with DENV, and produce more virus than LLC-MK2 cells when infected with the same viral titer. Therefore, C6/36 cells were used to detect DENV-2 titers in challenged mice by flow cytometry analysis as previously described [89].

### Antiviral activity of gossypol derivatives

A series of compounds (gossypol or its derivatives) were purchased from Timtec (Newark, DE, USA) and assessed by plaque assay for their inhibitory activity against infections of ZIKV and DENV [41, 71, 72, 74, 83]. Briefly, ZIKV (2.5×10^3^ PFU) was incubated with gossypol derivatives or gossypol, as control, at 37°C for 1 h. After removal of the unbound compounds by centrifugation (after addition of 3% PEG-6000), ZIKV was incubated with Vero E6 cells in 24-well plates at 37°C for 1 h. The cells were then washed with PBS, overlaid with DMEM containing 1% carboxymethyl cellulose and 2% FBS, and cultured at 37°C for 4–5 days, followed by being stained with 0.5% crystal violet. The inhibitory activity of gossypol derivatives or gossypol against DENV-1-4 was performed as described above, except that LLC-MK2 cells were used for infection, cultured at 37°C for 14–16 days, and then stained with 0.5% crystal violet. Plaques in each well were calculated, and IC_50_ of compounds for each compound was calculated using the CalcuSyn computer program [41, 71].

### In vitro cytotoxicity of gossypol derivatives

Cytotoxicity of gossypol derivatives was assessed in Vero E6 (for ZIKV) or LLC-MK2 cells (for DENV-1-4) using Cell Counting Kit-8 (CCK8, Sigma, St. Louis, MO, USA) according to the manufacturer’s instructions. Briefly, compounds at 2-fold serial dilutions were added to the respective cells (2.0×10^4^/well) pre-seeded in 96-well plates. The cells were cultured at 37°C in the presence of 5% CO_2_ for 3 days, lysed using cell lysis buffer, and then incubated with CCK8 solution, followed by measurement of absorbance at 450 nm (A450 value), using a microplate reader (Infinite F200PRO, Tecan, Morrisville, NC, USA). CC_50_ of compounds was calculated using the CalcuSyn program [41, 90, 91].

### Time-of-addition experiment

This experiment was carried out to detect potential inhibitory mechanisms of gossypol and its derivative ST087010 [41, 59, 92–97]. Briefly, Vero E6 cells (10^5^/well) and/or ZIKV were incubated in 24-well plates with or without the above compounds (15 μM) for 1 h before, 1 h after, or the same time during infection of ZIKV (the detailed procedures were described below and published previously [41]). The following six conditions of ZIKV infection were tested: (1) condition 1: pretreatment of the virus. ZIKV (PAN2016, 2.5×10^3^ PFU) was incubated with gossypol, ST087010, or DMSO control at 37°C for 1 h. After removal of the unbound compounds by centrifugation (after addition of 3% PEG-6000), ZIKV was incubated with cells at 37°C for 1 h. After incubation, the cells were cultured at 37°C for 4–5 days and stained with crystal violet, and plaques were visualized, based on which percent inhibition (% inhibition) of the compound was calculated. The plaque assay was performed following the standard protocol described above and before [84]. (2) condition 2: pretreatment of cells. Cells were preincubated with gossypol, ST087010, or DMSO control at 37°C for 1 h, and the unbound compounds were removed, followed by addition of ZIKV (PAN2016, 100 PFU) and incubation of cells at 37°C for 1 h. The unbound ZIKV was removed, and the cells were cultured and plaques calculated as in condition 1. (3) Condition 3: attachment. Cells were incubated with ZIKV (PAN2016, 300 PFU) at 4°C for 1 h in the presence of gossypol, ST087010, or DMSO control, which will allow ZIKV attachment, but not membrane fusion due to the energy requirement. After removal of the unbound ZIKV and compounds, the cells were cultured and plaques calculated as in condition 1. (4) Condition 4: cotreatment. Cells were infected with ZIKV (PAN2016, 100 PFU) at 37°C for 1 h in the presence of gossypol, ST087010, or DMSO control. After removal of the unbound virus and compounds, the cells were cultured and plaques were calculated as in condition 1. (5) condition 5: fusion. Cells were incubated with ZIKV (PAN2016, 300 PFU) at 4°C for 1 h for ZIKV attachment. After removal of the unbound ZIKV, the cells were incubated with gossypol, ST087010, or DMSO control at 37°C for 1 h for virus-cell membrane fusion. The unbound compounds were removed, and the cells were cultured and plaques calculated as in condition 1. (6) Condition 6: post-entry. Cells were incubated with ZIKV (PAN2016, 100 PFU) at 37°C for 1 h for ZIKV entry. After removal of the unbound ZIKV, the cells were incubated with gossypol, ST087010, or DMSO control at 37°C for 1 h. The unbound compounds were then removed, and the cells were cultured and calculated for plaques as in condition 1. Since variant conditions may affect the formation of plaques, the use of different PFUs can ensure that the number of plaques in the DMSO control group is about 100/well for each condition.

### Construction and expression of ZIKV NS2B-NS3 protease

Recombinant ZIKV NS2B-NS3 protease was constructed and expressed in an *E. coli* expression system [23, 98]. Briefly, genes encoding NS2B protein (residues 49-97) of ZIKV (GenBank accession no. NC_012532) were fused with the genes encoding NS3 protein (residues 1-185) through a linker (Gly4-Thr-Gly4). The amplified genes were cloned into pET-28b(+) expression vector (Novagen, Madison, Wisconsin, USA) containing a C-terminal His_6_ tag. The recombinant plasmid with correct sequences was transformed into *E.coli* for the expression of NS2B-NS3 protein. After addition of isopropyl-β–D-1-thiogalactopyranoside (IPTG, final concentration 1 mM) and culture of *E. coli* at 28°C for 12 h, the supernatants were collected for the purification of ZIKV NS2B-NS3 protein using Ni-NTA affinity chromatography, according to the manufacturer’s instructions (Qiagen, Qermantown, MD, USA).

### ELISA

ELISA was carried out to detect the binding between compounds (gossypol or its derivative ST087010) and ZIKV full-length E (Aviva Systems Biology, San Diego, CA, USA), EDIII (residues 298-409 in E protein containing a C-terminal human Fc tag) [83], or NS2B-NS3 protein [71, 83, 99, 100]. Briefly, ELISA plates were coated with each protein (1 μg/ml) at 4°C overnight and were then incubated with blocking buffer containing 2% fat-free milk in PBST (0.05% Tween-20) at 37°C for 2 h. The above compounds, or DMSO control, at serial dilutions were added to the plates and incubated at 37°C for 2 h. After three washes using PBST, the plates were incubated at 37°C for 2 h with ZIKV EDIII-specific human mAb ZKA64-LALA (0.5 μg/ml) (for binding to ZIKV full-length E or EDIII protein), or ZIKV NS2B-NS3-specific mouse sera (for binding to NS2B-NS3 protein). After further washes, the plates were incubated with horseradish peroxidase (HRP)-conjugated anti-human IgG-Fab (1:3,000, Abcam, Cambridge, MS, USA) or anti-mouse IgG (1:3,000, Sigma) antibody at 37°C for 1 h. The plates were incubated with substrate TMB (3,3′,5,5′-tetramethylbenzidine) for 1 min, and the reaction was stopped by addition of 1 N H_2_SO_4_. Absorbance at 450 nm (A450 values) was measured by an ELISA microplate reader. Percent binding (% binding) to the E, EDIII, or NS2B-NS3 protein was calculated in the presence or absence of serially diluted compounds using the formula ((1-(E/EDIII/NS2B-NS3-compound)/(E/EDIII/NS2B-NS3))×100), based on which EC_50_ was calculated using the CalcuSyn program [41, 71, 91].

ELISA was also used to determine the ability of ST087010 to inhibit the binding between ZIKV EDIII and EDIII-specific human mAbs (SMZAb5, ZKA64-LALA, ZV-67, or Z004) or EDI/II-specific human mAb (ZKA78) control [64]. This assay was carried out as described above, except that serially diluted ST087010, gossypol (positive control), or DMSO (negative control) was added to the plates in the presence of each mAb (0.5 μg/mL). The plates were then sequentially incubated with HRP-conjugated anti-human IgG-Fab antibody and TMB substrate, followed by detection of A450 values. Percent inhibition (% inhibition) of compounds was calculated using the formula ((1-(EDIII-mAb-compound)/(EDIII-mAb))×100), and IC_50_ was obtained using the CalcuSyn computer program [41, 91].

### SPR assay

The binding between gossypol derivative ST087010 and ZIKV EDIII or NS2B-NS3 protein was carried out by SPR using the Biacore BK system [92, 100] (GE Healthcare, Port Washington, NY, USA). Briefly, ZIKV EDIII protein or NS2B-NS3 protein was immobilized on a sensor chip (CM5) using the Amine Coupling Kit (GE Healthcare). Serially diluted ST087010 was added as analytes, and HBS-EP with 10% DMSO (10 mM HEPES, 150 mM NaCl, 3 mM EDTA, 0.05% Tween-20, 10% DMSO, pH 7.4) was used as a running buffer. Biacore BK evaluation software (version 1.1) was applied to analyze the data, and the curve was fitted with a 1:1 binding model.

### Inhibition of NS2B-NS3 protease activity

The ability of gossypol derivative ST087010 to inhibit NS2B-NS3 protease activity was carried out using a fluorescence-based enzymatic assay [49, 101]. Briefly, serially diluted ST087010, gossypol (positive control), or DMSO (negative control) was incubated with ZIKV NS2B-NS3 protein (1 μg/ml) at 37°C for 1 h in 96-well black micro-plates (Greiner Bio-One, Kremsmünster, Germany). Substrate (benzoyl-norleucinelysine-lysine-arginine 7-amino-4-methylcoumarine (Bz-Nle-Lys-Lys-Arg-AMC, 4 μM)) (Bachem, Torrance, CA, USA) was added and incubated for 10 min. Fluorescence intensity was then measured at 460 nm (with excitation at 355 nm) using a microplate reader (FLUOstar Omega, BMG Labtech, USA). A total of 10 mM Tris-HCl, 20% glycerol, 1 mM CHAPS, and 5% DMSO (pH 8.5) were used as reaction and dilution buffers. Percent inhibition (% inhibition) of protease activity was measured in the presence or absence of serially diluted compounds based on the formula ((1-(NS2B-NS3-substrate-compound)/(NS2B-NS3-substrate))×100). IC_50_ (concentration at 50% reduction of protease activity) was calculated using the CalcuSyn computer program based on the percent inhibition (% inhibition) of the compounds.

### Protective efficacy against ZIKV-caused lethal infection in adult *Ifnar1*^***−****/****−***^ mice

Gossypol derivative ST087010 was evaluated for its protective efficacy against ZIKV infection in ZIKV-susceptible *Ifnar1*^***−****/****−***^ mice [71, 72, 83, 102]. Briefly, 7–8-week-old male mice were used, and two separate experiments were performed. In experiment 1, groups of 6 mice were i.p. injected with ST087010 or gossypol (control) (20 mg/kg of body weight, 200 μl/mouse), or DMSO (negative control), 12 h before and 6, 24, and 48 h after infection. These mice were i.p. infected with ZIKV (human strain R103451, 200 PFU/mouse) and observed daily for weight changes and survival until 21 dpi. In experiment 2, groups of 5 mice were i.p. injected with ST087010, gossypol, or DMSO, as described above, and then i.p. infected with ZIKV (human strain PAN2016, 200 PFU/mouse). Five days later, these mice were sacrificed, and their tissues, including the heart, testis, eye, kidney, and brain, were collected for detection of viral titers by plaque assay, or apoptosis by immunofluorescence staining. Mice losing 20% of initial weight with severe symptoms, including hind limb weakness and paralysis, or 25% of initial weight, were humanely euthanized with an overdose isoflurane in a Bell Jar, followed by cervical dislocation.

Plaque assay was used to detect viral titers in ZIKV-infected tissues. Briefly, tissues were homogenized with cold culture medium (DMEM + 2% FBS) and then centrifuged (2000 *g* at 4°C for 10 min). Supernatants were serially diluted to infect Vero E6 cells in 24-well plates. ZIKV titers in tissues were measured from ~40 mg of samples; thus, the detection limit was about 25 PFU/g of tissues.

### Safety profile in pregnant *Ifnar1*^***−****/****−***^ mice and fetuses

Gossypol derivative ST087010 was assessed for its safety profiles in pregnant *Ifnar1*^***−****/****−***^ mice and their fetuses [103]. Briefly, groups of 5 pregnant mice (8–12-week-old, E12-14) were i.p. injected with ST087010 (20 or 40 mg/kg of body weight, 200 μl/mouse), or DMSO (negative control), daily for 4 continuous days. Mothers and pups at various prenatal and/or postnatal time points were observed for weight changes daily. Sera collected before and 4 h and 1, 3, and 5 days after the last injection were measured for ALT and creatinine levels using ALT and Creatinine Assay kits (Sigma). Two mothers and their pups (at 3-week-old) were sacrificed; their liver, spleen, kidney, and brain tissues sectioned and then assessed for histopathological changes by H&E staining.

### Protective efficacy against ZIKV vertical transmission in pregnant *Ifnar1*^***−****/****−***^ mice

Gossypol derivative ST087010 was evaluated for its protective efficacy against ZIKV-caused fetal damage and death in pregnant *Ifnar1*^***−****/****−***^ mice [77, 97]. Briefly, groups of 5 pregnant mice (10–12-week-old, E12-14) were i.p. injected with ST087010 (20 mg/kg of body weight, 200 μl/mouse), or DMSO (negative control), 12 h before and 6, 24, and 48 h after i.p. infection with ZIKV (human strain R116265, 10^3^ PFU). Five days post-infection, mice were sacrificed; viral titers were assessed using plaque assay in the sera, placenta, fetal brain, and amniotic fluid, and uteri and fetuses were assessed for morphological and size changes. The placenta was also assessed for apoptosis and viral replication by immunofluorescence staining, as described below.

### Protective efficacy against DENV infection in *Ifnar1*^***−****/****−***^ mice

Gossypol derivative ST087010 was evaluated for its protective efficacy against DENV-2 infection in susceptible *Ifnar1*^***−****/****−***^ mice [104–107]. Briefly, 3–4-week-old male mice were i.p. treated with ST087010, gossypol control (20 mg/kg of body weight, 200 μl/mouse), or DMSO (negative control) 12 h before and 6, 24 and 48 h after infection with DENV-2 human strain (V594, 2×10^6^ PFU/mouse). Three days after infection, sera and tissues, including the brain, kidney, and heart, were collected and assessed for DENV infection using a flow cytometry assay, as described below. Tissues were freshly homogenized with cold culture medium (EMEM + 2% FBS) and centrifuged at 4°C and 2000 *g* for 10 min. Serially diluted tissue supernatants and sera were added to C6/36 cells, as described below. DENV titers in tissues or sera were assessed from ~20 mg of tissue samples, or 25 μl of sera, and used for subsequent flow cytometry analysis.

### Flow cytometry assay

Flow cytometry assay was carried out to analyze DENV-2 titers in the supernatants of homogenized tissues and sera from DENV-2-infected mice collected above [89, 108, 109]. Briefly, homogenized tissue supernatants or sera were added to C6/36 cells (5×10^5^ cells/well) pre-seeded in 24-well plates which the culture medium was removed, and the cells were incubated for 1 h (28°C, 5% CO_2_). After incubation, the cells were washed with PBS, incubated with EMEM containing 2% FBS, and then cultured for 3 days as described above. After further removal of medium, the cells were digested, washed with PBS, and resuspended in FIX/PERM solution (Tonbo Biosciences, San Diego, CA, USA), followed by incubation at 4°C for 1 h in the dark. The cells were then sequentially incubated with mouse anti-flavivirus mAb 4G2 (2 μg/ml) at 37°C for 1 h and FITC-labeled anti-mouse IgG (0.5 mg/ml, BioLegend, San Diego, CA, USA) at 37°C for 30 min, followed by analysis using a FACScan flow cytometer and Summit software. Viral titers (infectious units/ml or infectious units/g) were calculated using the formula ((% of infected cells)×(total number of cells)×(dilution factor)/(amount of inoculum added to cells)) [89].

### Immunofluorescence staining

Immunofluorescence staining was carried out to detect ZIKV and caspase-3 signals in ZIKV-infected mouse tissues [72, 110–112]. Briefly, tissues were fixed in 4% formaldehyde, embedded in paraffin, and sectioned. The tissue sections were deparaffinized, fixed, and permed using the FIX and PERM Cell Permeabilization Kit (Thermo Fisher Scientific, Waltham, MA, USA). After blocking with 5% BSA, the tissue slides were incubated at 37°C 2 h with ZIKV EDIII-specific human mAb (ZV-67, 1:100, Absolute Antibody, Boston, MA, USA), or rabbit anti-active caspase-3 antibody (1:100, Abcam). The slides were washed with PBS and incubated for 30 min with anti-human FITC antibody (1:100, for ZIKV), or anti-rabbit Alexa Fluor 647 antibody (1:100, for caspase-3). The slides were then counterstained for nuclei, using DAPI (300 nM, Thermo Fisher Scientific, Waltham, MA, USA) for 5 min, and mounted in VectaMount Permanent Mounting Medium (Vector Laboratories, CA, USA). The slides were analyzed using a confocal microscope (Zeiss LSM 880) and ZEN software, and fluorescent signals were quantified by ImageJ software.

### Statistical analysis

The data are presented as mean plus s.e.m. Statistical significance among different groups was analyzed using Student’s two-tailed *t*-test and GraphPad Prism 7 Statistical Software. *, **, and *** indicate *P* < 0.05, *P* <0.01, and *P* < 0.001, respectively.

## Supplementary Information

**Additional file 1: Figure S1.** Alignment of amino acid sequences of NS2B-NS3 proteins of 10 ZIKV strains and DENV-1-3 human strains. Description: Schematic maps of ZIKV polyprotein and ZIKV NS2B-NS3 proteins, as well as alignment of amino acid residues, are shown. **Table S1.** In vitro inhibitory activity of gossypol and 16 derivatives against infection of ZIKV (PAN2016 strain).

## Data Availability

All data generated and/or analyzed during the current study are included in this published article and its supplementary information files.

## Abbreviations

ALT: Alanine transaminase
C: Capsid
CC_50_: 50% cytotoxic concentration
DAPI: 4′,6-Diamidino-2-phenylindole
DENV: Dengue virus
DHF: Dengue hemorrhagic fever
DMEM: Dulbecco’s modified Eagle’s medium
DSS: Dengue shock syndrome
E: Envelope
EC_50_: 50% effective concentration
EDI: E Protein domain I
EMEM: Eagle’s Minimal Essential Medium
ER: Endoplasmic reticulum
H&E: Hematoxylin and eosin staining
HRP: Horseradish peroxidase
IC_50_: 50% inhibitory concentration
*Ifnar1*^***−****/****−***^: Interferon-α/β receptor-deficient
IPTG: Isopropyl-β–D-1-thiogalactopyranoside
*K*_D_: Equilibrium dissociation constant
NS: Nonstructural proteins
PFU: Plaque-forming unit
prM/M: Pre-membrane/membrane
SI: Selectivity index
SPR: Surface plasmon resonance
TMB: 3,3′,5,5′-Tetramethylbenzidine
ZIKV: Zika virus
