# Supplementary material for: A gossypol derivative effectively protects against Zika and dengue virus infection without toxicity
Source: BMC Biol. 2022 Jun 15;20:143. doi: 10.1186/s12915-022-01344-w (PMC9202104; doi:10.1186/s12915-022-01344-w)
Supplement: Supplementary file 1 — Additional file 1: Figure S1. Alignment of amino acid sequences of NS2B-NS3 proteins of 10 ZIKV strains and DENV-1-3 human strains. Description: Schematic maps of ZIKV polyprotein and ZIKV NS2B-NS3 proteins, as well as alignment of amino acid residues, are shown. Table S1. In vitro inhibitory activity of gossypol and 16 derivatives against infection of ZIKV (PAN2016 strain). [file 12915_2022_1344_MOESM1_ESM.pdf]

Supplementary Figure S1

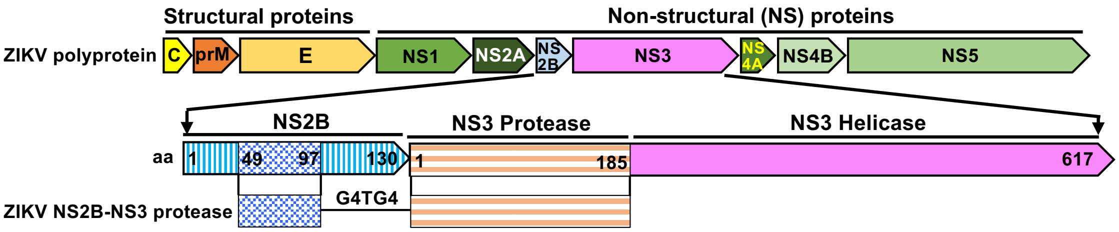


```
R116265         SWPPSEVLTAVGLICALAGGFAKADIEMAGPMAAVGLLIVSYVVSGKSVDMYIERAGDIT    60
PAN2016         SWPPSEVLTAVGLICALAGGFAKADIEMAGPMAAVGLLIVSYVVSGKSVDMYIERAGDIT    60
PAN2015         SWPPSEVLTAVGLICALAGGFAKADIEMAGPIAAVGLLIVSYVVSGKSVDMYIERAGDIT    60
FLR             SWPPSEVLTAVGLICALAGGFAKADIEMAGPMAAVGLLIVSYVVSGKSVDMYIERAGDIT    60
R103451         SWPPSEVLTAVGLICALAGGFAKADIEMAGPMAAVGLLIVSYVVSGKSVDMYIERAGDIT    60
PRVABC59        SWPPSEVLTAVGLICALAGGFAKADIEMAGPMAAVGLLIVSYVVSGKSVDMYIERAGDIT    60
PLCal_ZV        SWPPSEVLTAVGLICALAGGFAKADIEMAGPMAAVGLLIVSYVVSGKSVDMYIERAGDIT    60
IbH 30656       SWPPSEVLTAVGLICALAGGFAKADIEMAGPMAAVGLLIVSYVVTGKSVDMYIERAGDIT    60
MEX 2-81        SWPPSEVLTAVGLICALAGGFAKADIEMAGPMAAVGLLIVSYVVSGKSVDMYIERAGDIT    60
MR 766          SWPPSEVLTAVGLICALAGGFAKADIEMAGPMAAVGLLIVSYVVSGKSVDMYIERAGDIT    60
DENV-1-V1792    SWPLNEGIMAVGIVSILSSLLKNDVPLAGPLIAGGMLIACYVISGSSADLSLEKAAEVS    60
DENV-2-V594     SWPLNEAVMAVGMVSILASSLLKNDIPMTGPLVAGGLLTVCYVLTGRSADLELERAADVK    60
DENV-3-V1043    SWPLNEGVMAVGLVSILASSLLRNDVPMAGPLVAGGLLIACYVITGTSADLTVEKAADIT    60

R116265         WEKDAEVTGNSPRLDVALDESGDFSLVEDDGPPMREIILKVVLMTICGMNPIAIPFAAGA   120
PAN2016         WEKDAEVTGNSPRLDVALDESGDFSLVEDDGPPMREIILKVVLMTICGMNPIAIPFAAGA   120
PAN2015         WEKDAEVTGNSPRLDVALDESGDFSLVEDDGPPMREIILKVVLMTICGMNPIAIPFAAGA   120
FLR             WEKDAEVTGNSPRLDVALDESGDFSLVEDDGPPMREIILKVVLMTICGMNPIAIPFAAGA   120
R103451         WEKDAEVTGNSPRLDVALDESGDFSLVEDDGPPMREIILKVVLMTICGMNPIAIPFAAGA   120
PRVABC59        WEKDAEVTGNSPRLDVALDESGDFSLVEDDGPPMREIILKVVLMTICGMNPIAIPFAAGA   120
PLCal_ZV        WEKDAEVTGNSPRLDVALDESGDFSLVEDDGPPMREIILKVVLMTICGMNPIAIPFAAGA   120
IbH 30656       WEKDAEVTGNSPRLDVALDESGDFSLVEEDGPPMREIILKVVLMAICGMNPIAIPFAAGA   120
MEX 2-81        WEKDAEVTGNSPRLDVALDESGDFSLVEDDGPPMREIILKVVLMTICGMNPIAIPFAAGA   120
MR 766          WEKDAEVTGNSPRLDVALDESGDFSLVEEDGPPMREIILKVVLMAICGMNPIAIPFAAGA   120
DENV-1-V1792    WEEEAEHSGASHNILVEVQDDGTMKIKDEERDDTLTILLKATLLAVSGVYPLSIPATLFV   120
DENV-2-V594     WEDQAEISGSSPILSITISEDGSMSIKNEEEEHTLTILIRTGLLVISGVFPVSIPITAAA   120
DENV-3-V1043    WEEEAEQTGVSHNLMITVDDDGTMRIKDDETENILTVLLKTALLIVSGVFPYSIPATLLV   120

R116265         WYVYVKTGKRSGALWDVPAPKEVKKGETTDGVYRVMTRRLLGSTQVGVGIMQEGVFHTMW   180
PAN2016         WYVYVKTGKRSGALWDVPAPKEVKKGETTDGVYRVMTRRLLGSTQVGVGVMQEGVFHTMW   180
PAN2015         WYVYVKTGKRSGALWDVPAPKEVKKGETTDGVYRVMTRRLLGSTQVGVGVMQEGVFHTMW   180
FLR             WYVYVKTGKRSGALWDVPAPKEVKKGETTDGVYRVMTRRLLGSTQVGVGVMQEGVFHTMW   180
R103451         WYVYVKTGKRSGALWDVPAPKEVKKGETTDGVYRVMTRRLLGSTQVGVGVMQEGVFHTMW   180
PRVABC59        WYVYVKTGKRSGALWDVPAPKEVKKGETTDGVYRVMTRRLLGSTQVGVGVMQEGVFHTMW   180
PLCal_ZV        WYVYVKTGKRSGALWDVPAPKEVKKGETTDGVYRVMTRRLLGSTQVGVGVMQEGVFHTMW   180
IbH 30656       WYVYVKTGKRSGALWDVPAPKEVKKGETTDGVYRVMTRRLLGSTQVGVGVMQEGVFHTMW   180
MEX 2-81        WYVYVKTGKRSGALWDVPAPKEVKKGETTDGVYRVMTRRLLGSTQVGVGIMQEGVFHTMW   180
MR 766          WYVYVKTGKRSGALWDVPAPKEVKKGETTDGVYRVMTRRLLGSTQVGVGVMQEGVFHTMW   180
DENV-1-V1792    WYFWQKKKQRSGVLWDTPSPPEVERAVLDDGIYRIMQRGLLGRSQVGVGVFQENVFHTMW   180
DENV-2-V594     WYLWEVRKQRAGVLWDVPSPPPVGKAELEDGAYRIKQRGIFGYSQIGAGVYKEGTFHTMW   180
DENV-3-V1043    WHTWQKQTQRSGVLWDVPSPPETQKAELEEGVYRIKQQGIFGKTQVGVGVQKEGVFHTMW   180

R116265         HVTKGSALRSGEGRLDPYWGDVKQDLVSYCGPWKLDAAWDGHSEVQLLAVPPGERARNIQ   240
PAN2016         HVTKGSALRSGEGRLDPYWGDVKQDLVSYCGPWKLDAAWDGHSEVQLLAVPPGERARNIQ   240
PAN2015         HVTKGSALRSGEGRLDPYWGDVKQDLVSYCGPWKLDAAWDGHSEVQLLAVPPGERARNIQ   240
FLR             HVTKGSALRSGEGRLDPYWGDVKQDLVSYCGPWKLDAAWDGHSEVQLLAVPPGERARNIQ   240
R103451         HVTKGSALRSGEGRLDPYWGDVKQDLVSYCGPWKLDAAWDGHSEVQLLAVPPGERARNIQ   240
PRVABC59        HVTKGSALRSGEGRLDPYWGDVKQDLVSYCGPWKLDAAWDGHSEVQLLAVPPGERARNIQ   240
PLCal_ZV        HVTKGSALRSGEGRLDPYWGDVKQDLVSYCGPWKLDAAWDGHSEVQLLAVPPGERARNIQ   240
IbH 30656       HVTKGAALRSGEGRLDPYWGDVKQDLVSYCGPWKLDAAWDGLSEVQLLAVPPGERAKNIQ   240
MEX 2-81        HVTKGSALRSGEGRLDPYWGDVKQDLVSYCGPWKLDAAWDGHSEVQLLAVPPGERARNIQ   240
MR 766          HVTKGAALRSGEGRLDPYWGDVKQDLVSYCGPWKLDAAWDGLSEVQLLAVPPGERARNIQ   240
DENV-1-V1792    HVTRGAVLMYQGKRLEPSWASVKKDLISYGGGWRLRGSWNTGEEVQVIAVEPGKNPKNVQ   240
DENV-2-V594     HVTRGAVLMHRGKRIEPSWADVKKDLISYGGGWKLEGEWKEGEEVQVLALEPGKNPRAVQ   240
DENV-3-V1043    HVTRGAVLTYNGKRLEPNWASVKKDLISYGGGWRLSAQWQKGEEVQVIAVEPGKNPKNFQ   240
```

```
R116265         TLPGIFKTKDGDIGAVALDYPAGTSGSPILDKCGRVIGLYGNGVVIKNGSYVSAITQGRR   300
PAN2016         TLPGIFKTKDGDIGAVALDYPAGTSGSPILDKCGRVIGLYGNGVVIKNGSYVSAITQGRR   300
PAN2015         TLPGIFKTKDGDIGAVALDYPAGTSGSPILDKCGRVIGLYGNGVVIKNGSYVSAITQGRR   300
FLR             TLPGIFKTKDGDIGAVALDYPAGTSGSPILDKCGRVIGLYGNGVVIKNGSYVSAITQGRR   300
R103451         TLPGIFKTKDGDIGAVALDYPAGTSGSPILDKCGRVIGLYGNGVVIKNGSYVSAITQGRR   300
PRVABC59        TLPGIFKTKDGDIGAVALDYPAGTSGSPILDKCGRVIGLYGNGVVIKNGSYVSAITQGRR   300
PLCal_ZV        TLPGIFKTKDGDIGAVALDYPAGTSGSPILDKCGRVIGLYGNGVVIKNGSYVSAITQGRR   300
IbH 30656       TLPGIFKTKDGDIGAVALDYPAGTSGSPILDKCGRVIGLYGNGVVIKNGSYVSAITQGKR   300
MEX 2-81        TLPGIFKTKDGDIGAVALDYPAGTSGSPILDKCGRVIGLYGNGVVIKNGSYVSAITQGRR   300
MR 766          TLPGIFKTKDGDIGAVALDYPAGTSGSPILDKCGRVIGLYGNGVVIKNGSYVSAITQGKR   300
DENV-1-V1792    TAPGTFKTPEGEVGAIALDFKPGTSGSPIVNREGKIVGLYGNGVVTTSGTYVSAIAQAKA   300
DENV-2-V594     TKPGLFKTNTGTIGAVSLDFSPGTSGSPIVDRKGKVVGLYGNGVVTRSGAYVSAIAQTEK   300
DENV-3-V1043    TMPGTFQTTTGEIGAIALDFKPGTSGSPIINREGKVVGLYGNGVVTKNGGYVSGIAQTNA   300

R116265         EEETPVECFEPSMLKKKQLTVLDLHPGAGKTRRVLPEIVREAIKTRLRTVILAPTRVVAA   360
PAN2016         EEETPVECFEPSMLKKKQLTVLDLHPGAGKTRRVLPEIVREAIKTRLRTVILAPTRVVAA   360
PAN2015         EEETPVECFEPSMLKKKQLTVLDLHPGAGKTRRVLPEIVREAIKTRLRTVILAPTRVVAA   360
FLR             EEETPVECFEPSMLKKKQLTVLDLHPGAGKTRRVLPEIVREAIKTRLRTVILAPTRVVAA   360
R103451         EEETPVECFEPSMLKKKQLTVLDLHPGAGKTRRVLPEIVREAIKTRLRTVILAPTRVVAA   360
PRVABC59        EEETPVECFEPSMLKKKQLTVLDLHPGAGKTRRVLPEIVREAIKTRLRTVILAPTRVVAA   360
PLCal_ZV        EEETPVECFEPSMLKKKQLTVLDLHPGAGKTRRVLPEIVREAIKTRLRTVILAPTRVVAA   360
IbH 30656       EEETPVECFEPSMLRKKQLTVLDLHPGAGKTRRVLPEIVREAIKKRLRTVILAPTRVVAA   360
MEX 2-81        EEETPVECFEPSMLKKKQLTVLDLHPGAGKTRRVLPEIVREAIKTRLRTVILAPTRVVAA   360
MR 766          EEETPVECFEPSMLKKKQLTVLDLHPGAGKTRRVLPEIVREAIKKRLRTVILAPTRVVAA   360
DENV-1-V1792    SQEGPLPEIEDEVFRKRNLTIMDLHPGSGKTRRYLPAIVREAIKRKLRTLILAPTRVVAS   360
DENV-2-V594     SIEDN-PEIEDDIFRKKRLTIMDLHPGAGKTKRYLPAIVREAIKRGLRTLILAPTRVVAA   359
DENV-3-V1043    EPDGPTPELEEEMFKKRNLTIMDLHPGSGKTRKYLPAIVREAIKRRLRTLILAPTRVVAA   360

R116265         EMEEALRGLPVRYMTTAVNVTHSGTEIVDLMCHATFTSRLLQPIRVPNYNLYIMDEAHFT   420
PAN2016         EMEEALRGLPVRYMTTAVNVTHSGTEIVDLMCHATFTSRLLQPIRVPNYNLYIMDEAHFT   420
PAN2015         EMEEALRGLPVRYMTTAVNVTHSGTEIVDLMCHATFTSRLLQPIRVPNYNLYIMDEAHFT   420
FLR             EMEEALRGLPVRYMTTAVNVTHSGTEIVDLMCHATFTSRLLQPIRVPNYNLYIMDEAHFT   420
R103451         EMEEALRGLPVRYMTTAVNVTHSGTEIVDLMCHATFTSRLLQPIRVPNYNLYIMDEAHFT   420
PRVABC59        EMEEALRGLPVRYMTTAVNVTHSGTEIVDLMCHATFTSRLLQPIRVPNYNLYIMDEAHFT   420
PLCal_ZV        EMEEALRGLPVRYMTTAVNVTHSGTEIVDLMCHATFTSRLLQPIRVPNYNLYIMDEAHFT   420
IbH 30656       EMEEALRGLPVRYMTTAVNVTHSGTEIVDLMCHATFTSRLLQPIRVPNYNLYIMDEAHFT   420
MEX 2-81        EMEEALRGLPVRYMTTAVNVTHSGTEIVDLMCHATFTSRLLQPIRVPNYNLYIMDEAHFT   420
MR 766          EMEEALRGLPVRYMTTAVNVTHSGTEIVDLMCHATFTSRLLQPIRVPNYNLYIMDEAHFT   420
DENV-1-V1792    EMAEALKGMPIRYQTTAVKSEHTGKEIVDLMCHATFTMRLLSPVRVPNYNMIIMDEAHFT   420
DENV-2-V594     EMEEALRGLPIRYQTPAIRAEHTGREIVDLMCHATFTMRLLSPVRVPNYNLIIMDEAHFT   419
DENV-3-V1043    EMEEALKGLPIRYQTTATKSEHTGREIVDLMCHATFTMRLLSPVRVPNYNLIIMDEAHFT   420

R116265         DPSSIAARGYISTRVEMGEAAAIFMTATPPGTRDAFPDSNSPIMDTEVEVPERAWSSGFD   480
PAN2016         DPSSIAARGYISTRVEMGEAAAIFMTATPPGTRDAFPDSNSPIMDTEVEVPERAWSSGFD   480
PAN2015         DPSSIAARGYISTRVEMGEAAAIFMTATPPGTRDAFPDSNSPIMDTEVEVPERAWSSGFD   480
FLR             DPSSIAARGYISTRVEMGEAAAIFMTATPPGTRDAFPDSNSPIMDTEVEVPERAWSSGFD   480
R103451         DPSSIAARGYISTRVEMGEAAAIFMTATPPGTRDAFPDSNSPIMDTEVEVPERAWSSGFD   480
PRVABC59        DPSSIAARGYISTRVEMGEAAAIFMTATPPGTRDAFPDSNSPIMDTEVEVPERAWSSGFD   480
PLCal_ZV        DPSSIAARGYISTRVEMGEAAAIFMTATPPGTRDAFPDSNSPIMDTEVEVPERAWSSGFD   480
IbH 30656       DPSSIAARGYISTRVEMGEAAAIFMTATPPGTRDAFPDSNSPIMDTEVEVPERAWSSGFD   480
MEX 2-81        DPSSIAARGYISTRVEMGEAAAIFMTATPPGTRDAFPDSNSPIMDTEVEVPERAWSSGFD   480
MR 766          DPSSIAARGYISTRVEMGEAAAIFMTATPPGTRDAFPDSNSPIMDTEVEVPERAWSSGFD   480
DENV-1-V1792    DPSSIAARGYISTRVGMGEAAAIFMTATPPGSVEAFPQSNAVIQDEERDIPERSWNSGYE   480
DENV-2-V594     DPASIAARGYISTRVEMGEAAGIFMTATPPGSRDPFPQSNAPIMDEEREIPERSWNSGHE   479
DENV-3-V1043    DPASIAARGYISTRVGMGEAAAIFMTATPPGTADAFPQSNAPIQDEERDIPERSWNSGNE   480

R116265         WVTDHSGKTVWFVPSVRNGNEIAACLTKAGKRVIQLSRKTFETEFQKTKHQEWDFVVTTD   540
PAN2016         WVTDHSGKTVWFVPSVRNGNEIAACLTKAGKRVIQLSRKTFETEFQKTKHQEWDFVVTTD   540
PAN2015         WVTDHSGKTVWFVPSVRNGNEIAACLTKAGKRVIQLSRKTFETEFQKTKHQEWDFVVTTD   540
FLR             WVTDHSGKTVWFVPSVRNGNEIAACLTKAGKRVIQLSRKTFETEFQKTKHQEWDFVVTTD   540
R103451         WVTDHSGKTVWFVPSVRNGNEIAACLTKAGKRVIQLSRKTFETEFQKTKHQEWDFVVTTD   540
PRVABC59        WVTDHSGKTVWFVPSVRNGNEIAACLTKAGKRVIQLSRKTFETEFQKTKHQEWDFVVTTD   540
PLCal_ZV        WVTDHSGKTVWFVPSVRNGNEIAACLTKAGKRVIQLSRKTFETEFQKTKHQEWDFVVTTD   540
IbH 30656       WVTDHSGKTIWFVPSVRNGNEIAACLTKAGKRVIQLSRKTFETEFQKTKNQEWDFVITTD   540
MEX 2-81        WVTDHSGKTVWFVPSVRNGNEIAACLTKAGKRVIQLSRKTFETEFQKTKHQEWDFVVTTD   540
MR 766          WVTDHSGKTVWFVPSVRNGNEIAACLTKAGKRVIQLSRKTFETEFQKTKNQEWDFVITTD   540
DENV-1-V1792    WITDFPGKTVWFVPSIKSGNDIANCLRKNGKRVIQLSRKTFDTEYQKTKNNDWDYVVTTD   540
DENV-2-V594     WVTDFKGKTVWFVPSIKAGNDIAACLRKNGKKVIQLSRKTFDSEYVKTRANDWDFVVTTD   539
DENV-3-V1043    WITDFAGKTVWFVPSIKAGNDIANCLRKNGKKVIQLSRKTFDTEYQKTKLNDWDFVVTTD   540
```

```
R116265        ISEMGANFKADRVIDSRRCLKPVIL-DG-ERVILAGPMPVTHASAAQRRGRIGRNPNKPG   598
PAN2016        ISEMGANFKADRVIDSRRCLKPVIL-DG-ERVILAGPMPVTHASAAQRRGRIGRNPNKPG   598
PAN2015        ISEMGANFKADRVIDSRRCLKPVIL-DG-ERVILAGPMPVTHASAAQRRGRIGRNPNKPG   598
FLR            ISEMGANFKADRVIDSRRCLKPVIL-DG-ERVILAGPMPVTHASAAQRRGRIGRNPNKPG   598
R103451        ISEMGANFKADRVIDSRRCLKPVIL-DG-ERVILAGPMPVTHASAAQRRGRIGRNPNKPG   598
PRVABC59       ISEMGANFKADRVIDSRRCLKPVIL-DG-ERVILAGPMPVTHASAAQRRGRIGRNPNKPG   598
PLCal_ZV       ISEMGANFKADRVIDSRRCLKPVIL-DG-ERVILAGPMPVTHASAAQRRGRIGRNPNKPG   598
IbH 30656      ISEMGANFKADRVIDSRRCLKPVIL-DG-ERVILAGPMPVTHASAAQRRGRIGRNPNKPG   598
MEX 2-81       ISEMGANFKADRVIDSRRCLKPVIL-DG-ERVILAGPMPVTHASAAQRRGRIGRNPNKPG   598
MR 766         ISEMGANFKADRVIDSRRCLKPVIL-DG-ERVILAGPMPVTHASAAQRRGRIGRNPNKPG   598
DENV-1-V1792   ISEMGANFRADRVIDPRRCLKPVILKDGPERVILAGPMPVTVASAAQRRGRIGRNHNKEG   600
DENV-2-V594    ISEMGANFRAERVIDPRRCMKPVILTDGEERVILAGPMPVTHSSAAQRRGRIGRNPKNEN   599
DENV-3-V1043   ISEMGANFKADRVIDPRRCLKPVILTDGPERVILAGPMPVTAASAAQRRGRVGRNPQKEN   600

R116265        DEYLYGGGCAETDEDHAHWLEARMLLDNIYLQDGLIASLYRPEADKVAAIEGEFKLRTEQ   658
PAN2016        DEYLYGGGCAETDEDHAHWLEARMLLDNIYLQDGLIASLYRPEADKVAAIEGEFKLRTEQ   658
PAN2015        DEYLYGGGCAETDEDHAHWLEARMLLDNIYLQDGLIASLYRPEADKVAAIEGEFKLRTEQ   658
FLR            DEYLYGGGCAETDEDHAHWLEARMLLDNIYLQDGLIASLYRPEADKVAAIEGEFKLRTEQ   658
R103451        DEYLYGGGCAETDEDHAHWLEARMLLDNIYLQDGLIASLYRPEADKVAAIEGEFKLRTEQ   658
PRVABC59       DEYLYGGGCAETDEDHAHWLEARMLLDNIYLQDGLIASLYRPEADKVAAIEGEFKLRTEQ   658
PLCal_ZV       DEYLYGGGCAETDEDHAHWLEARMLLDNIYLQDGLIASLYRPEADKVAAIEGEFKLRTEQ   658
IbH 30656      DEYMYGGGCAETDEDHAHWLEARMLLDNIYLQDGLIASLYRPEADKVAAIEGEFKLRTEQ   658
MEX 2-81       DEYLYGGGCAETDEDHAHWLEARMLLDNIYLQDGLIASLYRPEADKVAAIEGEFKLRTEQ   658
MR 766         DEYMYGGGCAETDEGHAHWLEARMLLDNIYLQDGLIASLYRPEADKVAAIEGEFKLRTEQ   658
DENV-1-V1792   DQYIYMGQPLNNDEDHAHWTEAKMLLDNINTPEGIIPALFEPEREKSAAVDGEYRLRGEA   660
DENV-2-V594    DQYIYMGEPLENDEDCAHWKEAKMLLDNINTPEGIIPSMFEPEREKVDAIDGEYRLRGEA   659
DENV-3-V1043   DQYIFTGQPLNNDEDHAHWTEAKMLLDNINTPEGIIPALFEPEREKSAAIDGEYRLKGES   660

R116265        RKTFVELMKRGDLPVWLAYQVASAGITYTDRRWCFDGTTNNTILEDSVPAEVWTRHGEKR   718
PAN2016        RKTFVELMKRGDLPVWLAYQVASAGITYTDRRWCFDGTTNNTIMEDSVPAEVWTRHGEKR   718
PAN2015        RKTFVELMKRGDLPVWLAYQVASAGITYTDRRWCFDGTTNNTIMEDSVPAEVWTRHGEKR   718
FLR            RKTFVELMKRGDLPVWLAYQVASAGITYTDRRWCFDGTTNNTIMEDSVPAEVWTRHGEKR   718
R103451        RKTFVELMKRGDLPVWLAYQVASAGITYTDRRWCFDGTTNNTILEDSVPAEVWTRHGEKR   718
PRVABC59       RKTFVELMKRGDLPVWLAYQVASAGITYTDRRWCFDGTTNNTIMEDSVPAEVWTRHGEKR   718
PLCal_ZV       RKTFVELMKRGDLPVWLAYQVASAGITYTDRRWCFDGTTNNTIMEDSVPAEVWTRYGEKR   718
IbH 30656      RKTFVELMKRGDLPVWLAYQVASAGITYTDRRWCFDGTTNNTIMEDSVPAEVWTKYGEKR   718
MEX 2-81       RKTFVELMKRGDLPVWLAYQVASAGITYTDRRWCFDGTTNNTILEDSVPAEVWTRHGEKR   718
MR 766         RKTFVELMKRGDLPVWLAYQVASAGITYTDRRWCFDGTTNNTIMEDSVPAEVWTKYGEKR   718
DENV-1-V1792   RKTFVELMRRGDLPVWLSYKVASEGFQYSDRRWCFDGERNNQVLEENMDVEIWTKEGERK   720
DENV-2-V594    RKTFVDLMRRGDLPVWLAYRVAAEGINYADRRWCFDGIKNNQILEENVEVEIWTKEGERK   719
DENV-3-V1043   RKTFVELMRRGDLPVWLAHKVASEGIKYTDRKWCFDGQRNNQILEENMDVEIWTKEGEKK   720

R116265        VLKPRWMDARVCSDHAALKSFKEFAAGKR   747
PAN2016        VLKPRWMDARVCSDHAALKSFKEFAAGKR   747
PAN2015        VLKPRWMDARVCSDHAALKSFKEFAAGKR   747
FLR            VLKPRWMDARVCSDHAALKSFKEFAAGKR   747
R103451        VLKPRWMDARVCSDHAALKSFKEFAAGKR   747
PRVABC59       VLKPRWMDARVCSDHAALKSFKEFAAGKR   747
PLCal_ZV       VLKPRWMDARVCSDHAALKSFKEFAAGKR   747
IbH 30656      VLKPRWMDARVCSDHAALKSFKEFAAGKR   747
MEX 2-81       VLKPRWMDARVCSDHAALKSFKEFAAGKR   747
MR 766         VLKPRWMDARVCSDHAALKSFKEFAAGKR   747
DENV-1-V1792   KLRPRWLDARTYSDPLALREFKEFAAGRR   749
DENV-2-V594    KLKPRWLDARIYSDPLALKEFKEFAAGRK   748
DENV-3-V1043   KLRPRWLDARTYSDPLALKEFKDFAAGRK   749
```

Supplementary Figure Legends

Figure S1. Alignment of amino acid (aa) sequences of NS2B-NS3 proteins (including residues 49-97 of NS2B proteins and residues 1-185 of NS3 proteins) of 10 Zika virus (ZIKV) strains and dengue virus (DENV)-1-3 human strains used in this study. Schematic maps of ZIKV polyprotein and ZIKV NS2B-NS3 proteins are shown on top of the alignment. Variable amino acid residues are highlighted in cyan. The alignment for DENV-4 (PR-06-65-740) is not included since its sequence is not available. The alignment was performed using the CLUSTAL V method of the MegAlign program (DNASTAR v7.1). C, capsid; prM, precursor of membrane; E, envelope; NS1, NS2A, NS2B, NS3, NS4A, NS4B, and NS5, non-structural proteins.

Supplementary Tables

Table S1. *In vitro* inhibitory activity of gossypol and 16 derivatives against infection of ZIKV (PAN2016 strain)[a]

Compound	PAN2016	Vero E6	
	IC_{50} (μM)	CC_{50} (μM)	SI
**ST069299**	**2.34 ± 0.06**	**25.92 ± 2.70**	**11.08**
ST004331	35.02 ± 0.35	42.26 ± 1.17	1.20
**ST005138**	**2.29 ± 0.01**	**22.82 ± 0.02**	**9.97**
**ST086273**	**4.93 ± 0.70**	**50.46 ± 3.36**	**10.23**
ST005135	34.96 ± 2.86	103.70 ± 2.20	2.97
ST087012	31.78 ± 0.37	68.76 ± 0.36	2.16
ST088397	18.34 ± 2.01	23.25 ± 0.51	1.27
**ST087010**	**3.17 ± 0.18**	**49.56 ± 1.83**	**15.63**
ST088399	18.96 ± 1.03	63.95 ± 1.53	3.37
ST004335	27.38 ± 2.50	73.91 ± 1.68	2.7
ST087009	30.11 ± 0.12	86.34 ± 0.21	2.87
ST086271	27.38 ± 2.50	78.73 ± 0.71	2.88
**ST092971**	**4.98 ± 0.01**	**72.13 ± 1.91**	**14.48**
ST087945	36.39 ± 2.07	76.13 ± 0.22	2.09
ST087018	37.98 ± 0.76	154.98 ± 1.21	4.08
ST087947	32.98 ± 2.11	52.17 ± 2.08	1.58
Gossypol	3.78 ± 0.30	14.49 ± 0.07	3.83

[a]50% inhibitory concentration (IC_{50}) and 50% cytotoxic concentration (CC_{50}) of 16 gossypol derivatives were assessed in Vero E6 cells, and gossypol was included as control. The five "hit" compounds are highlighted in bold. SI (selectivity index) was calculated based on the values of CC_{50}/IC_{50}. The data are presented as the mean ± standard error of the mean (s.e.m.) of duplicate wells. The experiments were repeated twice with similar results.
